# Genetic Aspects and Molecular Testing in Prostate Cancer: A Report from a Dutch Multidisciplinary Consensus Meeting

**DOI:** 10.1016/j.euros.2022.11.011

**Published:** 2023-01-25

**Authors:** Niven Mehra, Iris Kloots, Michiel Vlaming, Shafak Aluwini, Els Dewulf, Daniela E. Oprea-Lager, Henk van der Poel, Herman Stoevelaar, Derya Yakar, Chris H. Bangma, Elise Bekers, Roderick van den Bergh, Andries M. Bergman, Franchette van den Berkmortel, Steve Boudewijns, Winand N.M. Dinjens, Jurgen Fütterer, Tom van der Hulle, Guido Jenster, Leonie I. Kroeze, Michel van Kruchten, Geert van Leenders, Pim J. van Leeuwen, Wendy W.J. de Leng, R. Jeroen A. van Moorselaar, Walter Noordzij, Rogier A. Oldenburg, Inge M. van Oort, Irma Oving, Jack A. Schalken, Ivo G. Schoots, Ed Schuuring, Robert J. Smeenk, Ben G.L. Vanneste, Erik Vegt, André N. Vis, Kim de Vries, Peter-Paul M. Willemse, Maurits Wondergem, Margreet Ausems

**Affiliations:** a Department of Medical Oncology, Radboud UMC, Nijmegen, The Netherlands; b Division Laboratories, Pharmacy and biomedical Genetics, Department of Genetics, University Medical Centre Utrecht, Utrecht, The Netherlands; c Department of Radiation Oncology, UMCG, Groningen, The Netherlands; d Centre for Decision Analysis & Support, Ismar Healthcare NV, Lier, Belgium; e Department of Radiology & Nuclear Medicine, Amsterdam University Medical Centers, VU University, Amsterdam, The Netherlands; f Department of Urology, Netherlands Cancer Institute-Antoni van Leeuwenhoek, Amsterdam, The Netherlands; g Department of Urology, Amsterdam University Medical Centers, VU University, Amsterdam, The Netherlands; h Department of Radiology, UMCG, Groningen, The Netherlands; i Department of Radiology, Netherlands Cancer Institute-Antoni van Leeuwenhoek, Amsterdam, The Netherlands; j Department of Urology, Erasmus MC, Rotterdam, The Netherlands; k Department of Pathology, Netherlands Cancer Institute-Antoni van Leeuwenhoek, Amsterdam, The Netherlands; l Department of Urology, St. Antonius Ziekenhuis Nieuwegein, The Netherlands; m Department of Medical Oncology and Oncogenomics, Netherlands Cancer Institute-Antoni van Leeuwenhoek, Amsterdam, The Netherlands; n Department of Internal Medicine, Zuyderland MC, Sittard, The Netherlands; o Department of Medical Oncology, Bravis Hospital, Roosendaal, The Netherlands; p Department of Pathology, Erasmus MC, Rotterdam, The Netherlands; q Department of Medical Imaging, Radboud UMC, Nijmegen, The Netherlands; r Department of Medical Oncology, Leiden University Medical Center, Leiden, The Netherlands; s Department of Pathology, Radboud UMC, Nijmegen, The Netherlands; t Department of Medical Oncology, University Medical Centre Groningen, Groningen, The Netherlands; u Department of Pathology, UMC Utrecht, Utrecht, The Netherlands; v Department of Nuclear Medicine & Molecular Imaging, University Medical Center Groningen, Groningen, The Netherlands; w Department of Clinical Genetics, Erasmus MC, Rotterdam, The Netherlands; x Department of Urology, Radboud UMC, Nijmegen, The Netherlands; y Department of Internal Medicine, Ziekenhuis Groep Twente, Almelo, The Netherlands; z Department of Radiology & Nuclear Medicine, Erasmus MC, Rotterdam, The Netherlands; aa Department of Pathology, University Medical Center Groningen, Groningen, The Netherlands; bb Department of Radiation Oncology, Radboud UMC, Nijmegen, The Netherlands; cc Department of Radiation Oncology (MAASTRO), GROW - School for Oncology and Developmental Biology, Maastricht UMC, Maastricht, The Netherlands; dd Department of Human Structure and Repair, Ghent University Hospital, Ghent, Belgium; ee Department of Radiation Oncology, Ghent University Hospital, Ghent, Belgium; ff Department of Radiation Oncology, Erasmus MC, Rotterdam, The Netherlands; gg Department of Urology, Cancer Center, UMC Utrecht, Utrecht, The Netherlands; hh Department of Nuclear Medicine, Netherlands Cancer Institute-Antoni van Leeuwenhoek, Amsterdam, The Netherlands

**Keywords:** *BRCA1/2*, Genetic counselling, Germline genetic testing, Prostate cancer, Tumour genetic testing, DNA damage repair, Mismatch repair, Castration-resistant prostate cancer

## Abstract

**Background:**

Germline and tumour genetic testing in prostate cancer (PCa) is becoming more broadly accepted, but testing indications and clinical consequences for carriers in each disease stage are not yet well defined.

**Objective:**

To determine the consensus of a Dutch multidisciplinary expert panel on the indication and application of germline and tumour genetic testing in PCa.

**Design, setting, and participants:**

The panel consisted of 39 specialists involved in PCa management. We used a modified Delphi method consisting of two voting rounds and a virtual consensus meeting.

**Outcome measurements and statistical analysis:**

Consensus was reached if ≥75% of the panellists chose the same option. Appropriateness was assessed by the RAND/UCLA appropriateness method.

**Results and limitations:**

Of the multiple-choice questions, 44% reached consensus. For men without PCa having a relevant family history (familial PCa/*BRCA*-related hereditary cancer), follow-up by prostate-specific antigen was considered appropriate. For patients with low-risk localised PCa and a family history of PCa, active surveillance was considered appropriate, except in case of the patient being a *BRCA2* germline pathogenic variant carrier. Germline and tumour genetic testing should not be done for nonmetastatic hormone-sensitive PCa in the absence of a relevant family history of cancer. Tumour genetic testing was deemed most appropriate for the identification of actionable variants, with uncertainty for germline testing. For tumour genetic testing in metastatic castration-resistant PCa, consensus was not reached for the timing and panel composition. The principal limitations are as follows: (1) a number of topics discussed lack scientific evidence, and therefore the recommendations are partly opinion based, and (2) there was a small number of experts per discipline.

**Conclusions:**

The outcomes of this Dutch consensus meeting may provide further guidance on genetic counselling and molecular testing related to PCa.

**Patient summary:**

A group of Dutch specialists discussed the use of germline and tumour genetic testing in prostate cancer (PCa) patients, indication of these tests (which patients and when), and impact of these tests on the management and treatment of PCa.

## Take Home Message

A Dutch multidisciplinary expert panel favoured tumour-first testing in metastatic prostate cancer (PCa) to identify actionable variants. Consensus was reached to trigger cascade germline genetic testing in metastatic PCa patients having *BRCA1/2* tumour pathogenic variants.

## 1 Introduction

The role of germline and tumour genetic testing in the care of men with prostate cancer (PCa) is increasing. The detection of pathogenic alterations (single nucleotide variants/insertions and deletions/structural variants) can impact treatment decisions and future cancer screening recommendations, and may also identify additional cancer risks for the patient’s male and female blood relatives. Germline pathogenic/likely pathogenic variants ([L]PVs) are reported in 7–16% of men with metastatic PCa and most frequently observed in DNA damage repair (DDR) and DNA mismatch repair (MMR) genes [1], [2], [3]. The most common (L)PVs in DDR genes are *BRCA2*, *CHEK2*, *ATM*, and *BRCA1* [2]. In localised disease, germline (L)PVs are found in ∼4–6% of men with high-risk and in <5% of men with low-risk localised PCa [2], [4], [5]. Somatic (L)PVs in DDR genes occur in ∼23% of patients with metastatic castration-resistant PCa (mCRPC), mainly in *BRCA2* and *ATM* [6]. Defective MMR genes and/or high microsatellite instability (MSI-H) occurs in ∼3–8% of PCa patients [7], [8]. Three biomarker-driven therapies have been approved by the Food and Drug Administration and/or European Medicines Agency for mCRPC. These are immune checkpoint inhibitor pembrolizumab for patients with advanced MMR-deficient and/or MSI-H cancers, and the poly ADP-ribose polymerase inhibitors (PARPi) olaparib and rucaparib for mCRPC patients with DDR alterations, including canonical genes in homologous recombination repair (HRR).

Despite a broader acceptance of the importance of germline and tumour genetic testing in PCa management, the application of testing still varies, partly due to uncertainties on its clinical consequences in various disease stages and a lack of clear recommendations for clinical practice. Regarding tumour genetic testing, there is no reimbursement, centralisation, or recommendations on gene panel or tissue requirements, and timing of testing. In addition, (inter)national guidelines show variability in their recommendations for germline and tumour genetic testing (Supplementary material) [9], [10], [11], [12]. To attempt to provide clarity in areas of consensus and uncertainty, a Dutch consensus meeting was organised, aiming to collect the opinion of a multidisciplinary expert panel on several clinical scenarios that can be used to support practising physicians in their management of PCa patients.

## 2 Participants and methods

### 2.1 Set-up

The consensus meeting was set up by a multidisciplinary scientific committee and an advising methodologist. The approach combined elements from the Delphi method, Nominal Group Technique, and consensus development techniques [13].

### 2.2 Panel composition

The multidisciplinary panel consisted of representatives from the following specialities: urology (*N* = 10), medical oncology (*N* = 8), radiation oncology (*N* = 4), clinical genetics (*N* = 4), pathology (*N* = 2), clinical molecular biology (*N* = 4), radiology (*N* = 3), and nuclear medicine (*N* = 4). The selection criteria included clinical and scientific expertise in the field of PCa, geographic spread, and availability to participate in both voting rounds and the virtual consensus meeting.

### 2.3 Explorative survey

Based on a literature search and clinical expertise of the scientific committee members, an explorative survey was compiled including different clinical scenarios with questions related to the genetic and molecular landscape of PCa. Panellists were asked to complete the survey and provide suggestions for improvement. Before completion of the survey, panellists were asked to watch two pre-recorded presentations on genetics of PCa and molecular testing for PCa.

### 2.4 Consensus meeting

The first-round survey results were discussed during a virtual conference meeting (February 11, 2022) in light of the available evidence, and proposals for adaptations were made. The refined clinical scenarios and questions were sent out in a second survey, 1 wk after the virtual conference. This survey was based on 11 patient scenarios and contained 36 questions, 18 with a multiple-choice format and 18 with rating the appropriateness of options on a nine-point scale (1–3: inappropriate, 4–6: uncertain, and 7–9: appropriate). All questions included the option “can’t judge” in case the expert lacked experience for a specific question or felt unable to vote for any other reason. Participants were explicitly instructed to give their personal opinion in light of the available evidence and (inter)national guidelines, disregarding the costs of testing and other potential constraints.

### 2.5 Statistical analysis

For multiple-choice questions, strong agreement (consensus) and fair agreement were defined as the situation in which ≥75% or 50–74% of the panellists, respectively, chose the same option. If the option “can’t judge” was chosen, the answer was excluded from the agreement calculations.

Appropriateness was calculated using the mathematical rules typically applied in RAND/UCLA appropriateness method studies [14]. An option was considered appropriate if the median panel score was between 7 and 9, and inappropriate if the median was between 1 and 3, in the absence of disagreement. Disagreement was defined as the situation in which at least one-third of the panellists scored in each of the sections 1–3 and 7–9. All other outcomes were deemed “equivocal/uncertain”.

## 3 Results

### 3.1 No PCa diagnosis but familial PCa and/or family history of other cancers

In all scenarios (Supplementary material), follow-up by prostate-specific antigen (PSA) was considered appropriate and no further follow-up inappropriate (Table 1). Referral to a clinical geneticist for further genetic counselling was deemed appropriate when a first-degree relative had ovarian/breast cancer, but uncertain if only familial PCa was present (Table 1).

**Table 1:** Panel appropriateness outcomes for a man without a PCa diagnosis, and presence of familial PCa with or without other relevant family history of cancer (*N* = 39 panellists)

DRE = digital rectal examination; (L)PV = (likely) pathogenic variant; mpMRI = multiparametric magnetic resonance imaging; PCa = prostate cancer; PSA = prostate-specific antigen.

Appropriateness: green—appropriate (median score 7–9, no disagreement), red—inappropriate (median score 1–3, no disagreement), yellow—uncertain (median score 4–6 and/or disagreement).

(D) Disagreement: at least one-third of the scores in each of the sections 1–3 and 7–9.

Familial PCa: Three or more family members with PCa, or two or more family members with PCa diagnosed at ≤55 yr of age, or PCa in three generations within one branch of the family. In all cases: first- or second-degree family members and PCa with Gleason score ≥7 [11].

^a^Especially important for the female relatives of the man.

### 3.2 Low-risk PCa and family history of PCa

In these clinical scenarios (Supplementary material), the panellists considered tumour genetic testing inappropriate and germline testing appropriate only if a *BRCA2* germline (L)PV was present in the family (Table 2). Active surveillance (AS) was considered appropriate in most scenarios, but uncertain when the patient was a carrier of the *BRCA2* germline (L)PV that was identified in the patient's family (Table 2).

**Table 2:** Appropriateness outcomes for a man diagnosed with low-risk PCa (*N* = 39 panellists)

ECOG PS = Eastern Cooperative Oncology Group performance status; (L)PV = (likely) pathogenic variant; mHSPC = metastatic hormone-sensitive prostate cancer; PCa = prostate cancer; PSA = prostate-specific antigen.

Appropriateness: green—appropriate (median score 7–9, no disagreement), red—inappropriate (median score 1–3, no disagreement), yellow—uncertain (median score 4–6 and/or disagreement).

### 3.3 Nonmetastatic hormone-sensitive PCa

There was consensus that germline (85%) or tumour (81%) genetic testing should not be recommended for patients with nonmetastatic hormone-sensitive PCa (HSPC) not having a relevant family history of PCa or other cancers. Germline testing was considered appropriate for patients with a family history of relevant cancers and uncertain for those with a PCa family history. Tumour genetic testing was not recommended in these subgroups (Supplementary material).

### 3.4 Metastatic HSPC

In the absence of a relevant family history, scenarios for germline testing were deemed inappropriate for both the de novo and the recurrent setting in patients with M1a disease, visceral metastases (liver/lung), high-volume disease (bone), and low-volume disease. Tumour genetic testing was deemed inappropriate for de novo M1a HSPC and uncertain in all other scenarios (Supplementary material). If tumour genetic testing was performed, the panellists considered further germline testing appropriate in patients with high-volume mHSPC (CHAARTED criteria) having a *BRCA1/2* tumour (L)PV or a non-*BRCA1/2* tumour (L)PV (eg, *ATM*, *CHEK2*; Supplementary material). The panellists agreed that the choice of upfront treatment does not depend on the presence of a *BRCA2* tumour (L)PV in both de novo low-volume and high-volume disease (Table 3). In mHSPC patients for whom tumour genetic testing is recommended, a biopsy of a metastatic lesion was preferred over other options (Table 3).

**Table 3:** Panel results on questions regarding mHSPC (*N* = 39 panellists)

Question	Panellists (%) a	Can’t judge (%)
Does the presence of a *BRCA2* tumour (L)PV in a patient with de novo, low-volume mHSPC have an impact on the choice of your upfront treatment?		35.9
No	**88.0**	
Yes	12.0	
Does the presence of a tumour (L)PV in a non-*BRCA* gene (eg, *CHEK2*) in a patient with de novo, low-volume mHSPC have an impact on the choice of your upfront treatment?		35.9
No	**96.0**	
Yes	4.0	
Does the presence of a *BRCA2* tumour (L)PV in a patient with de novo, high-volume mHSPC have an impact on the choice of your upfront treatment?		35.9
No	**76.0**	
Yes	24.0	
In case a tumour genetic test is recommended for a patient with a primary diagnosis of mHSPC (de novo), then the following source of tissue is preferred:		23.1
Biopsy primary tumour	20.0	
Biopsy metastatic lesion	**80.0**	
In case a tumour genetic test is recommended for a patient with progressive disease following curative treatment, then the following source of tissue is preferred:		17.9
Archived tissue of primary tumour	3.1	
New biopsy of primary tumour if possible	9.4	
Biopsy of a newly diagnosed metastatic lesion	**87.5**	

(L)PV = (likely) pathogenic variant; mHSPC = metastatic hormone-sensitive prostate cancer.

The bold values represent statements for which ≥75% of the panellists chose the same option (consensus).

a Percentage of valid answers, that is, after the exclusion of the “can’t judge” category.

### 3.5 Metastatic CRPC

**Fig. 1:**
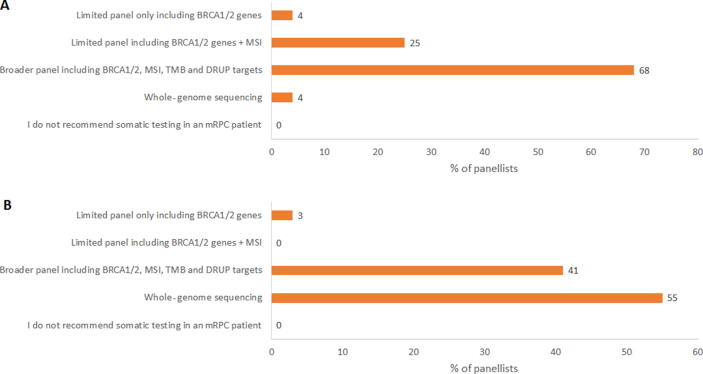
Panel outcomes on the most relevant gene panel to use for tumour genetic testing in a patient with mCRPC for (A) clinical decision-making in daily practice and (B) research purposes. DRUP = Drug Recovery Protocol (Dutch study investigating the efficacy of targeted therapy and immunotherapy, in patients with advanced/metastatic cancer, not eligible for standard treatment and having specific somatic mutations); mCRPC = metastatic castration-resistant prostate cancer; MSI = microsatellite instability; TMB = tumour mutation burden.

Germline testing was considered uncertain while tumour genetic testing was considered appropriate to identify actionable variants in a mCRPC patient (Table 4). Specific patient and disease characteristics hardly influenced the appropriateness of performing tumour genetic testing (Supplementary material). Further germline testing was deemed appropriate in mCRPC patients having a *BRCA1/2* tumour (L)PV (Table 4). For mCRPC patients fit enough to receive several therapy lines and not yet treated with novel hormonal agents (NHAs) or chemotherapy in the mHSPC setting, the opinions on the timing of tumour testing were dispersed: in routine practice, 35% of the panellists would recommend a tumour genetic test before any treatment for mCRPC, 26% after an NHA for mCRPC, 35% after an NHA and docetaxel for mCRPC, and 4% if no other treatment options are available (Supplementary material). In an experimental setting, the proportion of panellists recommending a tumour genetic test before any treatment for mCRPC was substantially higher (74%). Regarding the tissue source for tumour genetic testing, a new biopsy of a metastatic lesion was favoured over the other options (Table 4). For performing tumour genetic testing, no agreement was found for the minimum requirement of tumour percentage of the biopsy material and the biopsy age (Supplementary material). The recommendations for the gene panel composition differed between routine clinical practice (registered drugs) and experimental options, and larger panels would be preferred in experimental situations (Fig. 1). In relation to the required facilities for tumour genetic testing, 53% of the panellists recommended a hospital with a molecular tumour board and a clinical genetic laboratory, where 44% considered access to a molecular tumour board to be sufficient (Supplementary material). There was no consensus on when to initiate a targeted therapy or immunotherapy in an mCRPC patient with specific actionable (L)PVs (Table 5).

**Table 4:** Panel appropriateness outcomes in the mCRPC setting (*N* = 39 panellists)

(L)PV = (likely) pathogenic variant; mCRPC = metastatic castration-resistant prostate cancer.

No disagreement for all questions.

Appropriateness: green—appropriate (median score 7–9, no disagreement) and yellow—uncertain (median score 4–6 and/or disagreement).

**Table 5:** Panel results on questions regarding treatment choices for patients with mCRPC (*N* = 39 panellists)

Question	Panellists (%) a	Can’t judge (%)
When would you use a PARPi in a patient with mCRPC and a BRCA1/2 germline and/or tumour (L)PV?		53.8
After one line of NHA	27.8	
After one line of NHA and docetaxel	72.2	
After one line of NHA and two lines of chemotherapy	0	
Not as long as other treatment options are available	0	
When would you use an immune checkpoint inhibitor in a patient with mCRPC and an MMR (L)PV or MSI?		53.8
After one line of NHA and docetaxel	61.1	
After one line of NHA and two lines of chemotherapy	33.3	
I would not use an immune checkpoint inhibitor	5.6	
Do you recommend platinum-based chemotherapy to patients with mCRPC and a BRCA1/2 tumour and/or germline (L)PV before a possible treatment with a PARPi?		56.4
Yes, but only when naïve for NHA	5.9	
Yes, after ≥1 lines of NHA	17.6	
Yes, after ≥1 lines of NHA and 1 line of chemotherapy	23.5	
No	52.9	
Do you recommend platinum-based chemotherapy to patients with mCRPC and a BRCA1/2 tumour and/or germline (L)PV after progression with a PARPi?		59.0
Yes	**81.3**	
No	18.8	

(L)PV = (likely) pathogenic variant; mCRPC = metastatic castration-resistant prostate cancer; MMR = mismatch repair; MSI = microsatellite instability; NHA = novel hormonal agent; PARPi = poly ADP-ribose polymerase inhibitor.

The bold values represent statements for which ≥75% of the panellists chose the same option (consensus).

a Percentage of valid answers, that is, after the exclusion of the “can’t judge” category.

## 4 Discussion

### 4.1 No PCa diagnosis

For men without PCa but having a relevant family history, being either familial PCa or a positive family history of *BRCA*-related hereditary cancer (eg, breast, ovarian, and/or pancreatic cancer in first- or second-degree relative), follow-up by PSA alone was considered appropriate. This is in line with the current Dutch guidelines, recommending PCa screening for first-degree relatives of patients from families with familial PCa starting at 50 yr of age (or 5 yr before the age of the youngest diagnosed PCa patient in the family) or for *BRCA2* (L)PV carriers starting from 45 yr of age (or 5 yr before the age of the youngest diagnosed PCa patient in the family) [11]. In addition, the European Association of Urology guidelines recommend early PSA testing for well-informed men from 45 yr of age and having a family history of PCa, and for men carrying *BRCA2* (L)PVs from 40 yr of age [9]. The IMPACT study, evaluating the role of targeted PSA screening in men with *BRCA1/2* or MMR germline (L)PVs, supports the role of yearly PSA screening in men aged 40–69 yr with *BRCA2, MSH2*, and *MSH6* germline (L)PVs [15], [16]. Another study evaluated imaging- and PSA-based PCa screening in men aged 40–70 yr with *BRCA1/2* germline (L)PVs, and found that carriers <55 yr could benefit from initial multiparametric magnetic resonance imaging (mpMRI) screening [17]. However, 90% of patients had a Jewish founder (L)PV, and therefore results cannot be generalised to other ethnic groups. Multiparametric MRI is not recommended as a screening tool by international guidelines [9], with additional research needed to underscore the value of mpMRI in patients with germline (L)PVs in diverse ethnic backgrounds, and in those with elevated and nonelevated PSA levels. At present, the panel deemed follow-up by mpMRI inappropriate/uncertain in the queried clinical scenarios. Referral to a clinical geneticist for further genetic counselling was considered uncertain in case of familial PCa, but appropriate if a positive family history of breast/ovarian cancer was present. The current Dutch guidelines do not recommend germline testing for families with familial PCa only [11]. These outcomes also reflect that collecting detailed family history, not limited to PCa, is key.

### 4.2 Nonmetastatic HSPC

AS is a recommended management strategy for patients with low-risk localised PCa [9]. For the scenarios with low-risk PCa and PCa family history, the outcome for AS was appropriate but changed to uncertain when the patient had a *BRCA2* germline (L)PV. Germline (L)PVs in *BRCA2* and *ATM* seem to be associated with an aggressive phenotype, lethal PCa, and earlier age at death and shorter disease-specific survival [18], [19]. Data showed that among men undergoing AS, grade reclassification was associated with *BRCA1/2* and *ATM* germline (L)PVs [20]. This association was strongest for men with *BRCA2* (L)PVs. *BRCA2* carriers had a five-fold higher risk of reclassification to Gleason grade group 3 after diagnosis of Gleason grade group 1 versus noncarriers. Although long-term outcomes of AS for low-risk PCa among *BRCA2* carriers are not available, AS may be feasible for these patients, but careful monitoring is advised. Consensus was reached that germline and/or tumour genetic testing should not be considered in patients diagnosed with localised/locally advanced PCa who lack a family history of PCa or other cancers (Supplementary material), which is in contrast with the National Comprehensive Cancer Network guideline recommendations (Supplementary material) [10]. No pathological factor was considered appropriate to perform germline and/or tumour genetic testing for patients with nonmetastatic HSPC. However, germline (L)PVs, especially in *BRCA2*, *ATM*, and *MSH2*, seem to be associated with PCa in grade group 5 [5]. Another study found that a Gleason score of ≥8 was associated with DDR germline (L)PVs [21]. Several retrospective studies showed an association between the presence of intraductal or cribriform pathology, and germline (L)PVs in DDR genes, mainly *BRCA2* [22].

### 4.3 Metastatic PCa

Although generally recommended by international guidelines [9], [10], [12], the panel considered a more limited role for germline testing in patients diagnosed with metastatic PCa. It was particularly recommended when *BRCA1/2* or non-*BRCA1/2* tumour (L)PV (eg, *ATM*, *CHEK2*) were identified (Supplementary material). A germline-focussed analysis following tumour sequencing has multiple advantages in the metastatic PCa setting [23]. More than half actionable DDR mutations will be missed by germline testing alone, and therefore germline testing should never substitute for tumour testing, when the goal is providing access to precision medicine. Only patients with tumour-detected pathogenic variants of potential germline origin can be referred for genetic counselling and subsequent germline testing. This improves efficiency as there is a lack of genetic counsellors and geneticists. We also acknowledge the importance of increasing awareness about the personal and family characteristics that are related to possible germline pathogenic variants. To improve germline genetic testing in patients who fulfil clinical genetic criteria for germline genetic testing, urologists and oncologists may provide pretest genetic counselling and order germline genetic tests (the so-called mainstream genetic testing pathway) [21]. Several initiatives around the world currently assess the feasibility of this pathway in PCa care. It should be noted that in 7–10% of patients with established germline (L)PVs, these variants were missed in tumour sequencing [24], [25]. This warrants a patient-specific consideration of germline testing in patients with a negative tumour test, when a tumour-first pathway is applied.

The panellists preferred newly obtained biopsies over archived diagnostic tissue of the primary tumour as a source for tumour genetic testing (Supplementary material). In the phase III PROfound study, next-generation sequencing (NGS) results were acquired in 58% of the samples, and were more frequently achieved for newly obtained versus archival and for metastatic versus primary tumour samples [26]. However, nearly half of mCRPC patients have bone-predominant disease, and biopsies of bone lesions can be a technically challenging procedure [27]. With regard to the assessment of the full spectrum of actionable alterations, the tumour content impacts the ability to identify homozygous deletions and biallelic inactivation/loss of heterozygosity. Opinions on the minimum needed tumour content, the minimum tissue sample age, which gene panel to use, and the timing for somatic testing varied considerably, and no consensus was obtained. Owing to a genetic drift partly due to selective pressure from anticancer therapies, tumour (L)PVs may evolve over time, questioning the timing to perform tumour genetic testing and the choice of tissue sample [28], [29]. The chance of obtaining an NGS result declined with increasing sample age, mainly because of failure to extract sufficient DNA, but approximately half of the samples aged >10 yr yielded results [26]. Another study found that primary prostate tissue accurately reflects the status of the most common actionable DDR gene alterations with an overall concordance with metastatic tissue of 84% [30]. In another matched series, also minimal discordance between hormone-naïve and mCRPC biopsies was seen in relevant DDR genes [31]. However, for infrequent druggable alterations, a metastatic tissue sample might still be more accurate in identifying actionable (L)PVs and resistance mechanisms.

In patients with de novo mHSPC, presence of a *BRCA2* tumour (L)PV would influence the choice of upfront treatment in only a minority of panellists (Supplementary material). The panellists mostly opted for more aggressive treatment, mainly addition of an NHA, although some would consider triplet therapy with an NHA and docetaxel for high-volume disease. Prior taxane therapy was not mandated in the PROfound study, with unplanned subgroup analyses showing efficacy for olaparib independent of taxane use [32]. There was fair agreement between the panellists (72%) to offer a PARPi following one line of NHA and one line of chemotherapy. Opinions on the timing to perform tumour genetic testing in daily clinical practice for an mCRPC patient were dispersed (Supplementary material). When patient allocation into precision medicine trials is necessary, there was fair agreement (74%) that tumour genetic testing should ideally be performed in treatment-naïve mCRPC (Supplementary material). According to a recent study, 45.5% of Dutch patients harbour one or more actionable alterations in genes as per the ESMO Precision Medicine Working Group recommendation, with >60% of patients allocated within a precision medicine trial, including the Drug Rediscovery Protocol (DRUP) trial running in over 30 Dutch centres [33]. As targeted therapy and immunotherapy within this trial can be administered only after NHAs and taxanes, some panellists believed that many patients would not be suitable study candidates when tested in end-stage (mCRPC) disease. In those cases, tumour genetic testing should be avoided.

### 4.4 Limitations

As some topics discussed lack scientific evidence, recommendations are partly opinion based. Another limitation concerns the small number of experts per discipline.

## 5 Conclusions

The identification of germline and/or tumour (L)PVs could have a profound impact on the management of PCa. The results of this multidisciplinary consensus study may provide further guidance to practising clinicians.

  ***Author contributions*:** Niven Mehra had full access to all the data in the study and takes responsibility for the integrity of the data and the accuracy of the data analysis.

  *Study concept and design*: Aluwini, Ausems, Dewulf, Mehra, Oprea-Lager, van der Poel, Stoevelaar, Yakar.

*Acquisition of data*: Aluwini, Ausems, Dewulf, Mehra, Oprea-Lager, van der Poel, Stoevelaar, Yakar.

*Analysis and interpretation of data*: Aluwini, Ausems, Dewulf, Kloots, Mehra, Oprea-Lager, van der Poel, Stoevelaar, Vlaming, Yakar.

*Drafting of the manuscript*: Ausems, Dewulf, Mehra, Stoevelaar.

*Critical revision of the manuscript for important intellectual content*: Aluwini, Ausems, Kloots, Oprea-Lager, van der Poel, Vlaming, Yakar.

*Statistical analysis*: Stoevelaar.

*Obtaining funding*: Aluwini, Mehra, Oprea-Lager, van der Poel, Yakar.

*Administrative, technical, or material support*: Dewulf, Stoevelaar.

*Supervision*: Stoevelaar.

*Other*: Consensus ratings, review statements, and review manuscript: Bangma, Bekers, van den Bergh, Bergman, van den Berkmortel, Boudewijns, Dinjens, Fütterer, van der Hulle, Jenster, Kroeze, van Kruchten, van Leenders, van Leeuwen, de Leng, van Moorselaar, Noordzij, Oldenburg, van Oort, Oving, Schalken, Schoots, Schuuring, Smeenk, Vanneste, Vegt, Vis, de Vries, Willemse, Wondergem.

  ***Financial disclosures:*** Niven Mehra certifies that all conflicts of interest, including specific financial interests and relationships and affiliations relevant to the subject matter or materials discussed in the manuscript (eg, employment/affiliation, grants or funding, consultancies, honoraria, stock ownership or options, expert testimony, royalties, or patents filed, received, or pending), are the following: Niven Mehra: advisory role (compensated and institutional) at Janssen, Astellas, AstraZeneca, JNJ, MSD, Pfizer, and Roche; research support (institutional, no financial interest) from Astellas, AstraZeneca, BMS, Janssen, and Pfizer; and research support (personal, no financial interest) from Janssen. Shafak Aluwini: consultancy fee from Astellas. Els Dewulf: employee of Ismar Healthcare NV. Iris Kloots: none. Daniela E. Oprea-Lager: institutional consultancy fee from Astellas, during the conduct of the study, and two unrestricted grants from Janssen in 2020 and 2022, outside the submitted work. Henk van der Poel: consultancy fee from Astellas. Michiel Vlaming: none. Derya Yakar: consultancy fee from Astellas. Herman Stoevelaar: partner in Ismar Healthcare NV. Margreet Ausems: consultancy fee from Astellas. Andries M. Bergman: consultancy fees from Astellas, Bayer, and Jansen. Inge M. van Oort: in advisory boards of MSD and AstraZeneca. Ed Schuuring: performed lectures for Bio-Rad, Novartis, Roche, Biocartis, Illumina, Pfizer, AstraZeneca, and Agena Bioscience; consultant in advisory boards for AstraZeneca, Roche, Pfizer, Novartis, Bayer, BMS, Amgen, BioCartis, Illumina, Agena Bioscience, MSD/Merck, and GSK; and research grants from AstraZeneca, Pfizer, Biocartis, Agena Bioscience, BMS, Bio-Rad, Roche, and Boehringer Ingelheim, all transferred to UMCG account.

  ***Funding/Support and role of the sponsor*:** The consensus study was funded by Astellas Pharma B.V., but this company had no influence on the content in any stage of the process.

  ***Acknowledgments*:** The authors are grateful to Ismar Healthcare NV for supporting the consensus study and for editorial assistance during the manuscript preparation. Panel members (in alphabetic order by speciality): clinical genetics: Margreet Ausems (UMC Utrecht), Marleen Kets (Radboudumc Nijmegen), Rogier Oldenburg (Erasmus MC Rotterdam), Michiel Vlaming (UMC Utrecht); clinical molecular biology: Winand Dinjens (Erasmus MC Rotterdam), Leonie Kroeze (Radboudumc Nijmegen), Wendy de Leng (UMC Utrecht), Ed Schuuring (UMC Groningen); medical oncology: André Bergman (NKI-AVL Amsterdam), Franchette van den Berkmortel (Zuyderland Sittard-Geleen), Steve Boudewijns (Bravis Oncologie Centrum Roosendaal), Tom van der Hulle (LUMC Leiden), Iris Koots (Radboudumc Nijmegen), Michel van Kruchten (UMC Groningen), Niven Mehra (Radboudumc Nijmegen), Irma Oving (ZGT Almelo); nuclear medicine: Walter Noordzij (UMC Groningen), Daniela Oprea-Lager (Amsterdam UMC), Erik Vegt (Erasmus MC Rotterdam), Maurits Wondergem (NKI-AVL Amsterdam); pathology: Elise Bekers (NKI-AVL Amsterdam), Geert van Leenders (Erasmus MC Rotterdam); radiology: Jurgen Fütterer (Radboudumc Nijmegen), Ivo Schoots (Erasmus MC Rotterdam), Derya Yakar (UMC Groningen and NKI-AVL Amsterdam); radiation oncology: Shafak Aluwini (UMC Groningen), Robert-Jan Smeenk (Radboudumc Nijmegen), Ben Vanneste (MAASTRO Maastricht), Kim de Vries (Erasmus MC Rotterdam); urology: Chris Bangma (Erasmus MC Rotterdam), Roderick van den Bergh (St. Antonius Ziekenhuis Nieuwegein), Guido Jenster (Erasmus MC Rotterdam), Pim van Leeuwen (NKI-AVL Amsterdam), Jeroen van Moorselaar (Amsterdam UMC), Inge van Oort (Radboudumc Nijmegen), Henk van der Poel (NKI-AVL Amsterdam and Amsterdam UMC), Jack Schalken (Radboudumc Nijmegen), André Vis (Amsterdam UMC), Peter-Paul Willemse (UMC Utrecht). Speakers of pre-recorded presentations: Iris Kloots and Michiel Vlaming. Scientific Committee: Shafak Aluwini, Margreet Ausems, Niven Mehra, Daniela Oprea-Lager, Henk van der Poel, and Derya Yakar.

## References

[1] Boyle J.L., Hahn A.W., Kapron A.L. (2020). Pathogenic germline DNA repair gene and HOXB13 mutations in men with metastatic prostate cancer. JCO Precis Oncol.

[2] Pritchard C.C., Mateo J., Walsh M.F. (2016). Inherited DNA-repair gene mutations in men with metastatic prostate cancer. N Engl J Med.

[3] Castro E., Romero-Laorden N., Del Pozo A. (2019). PROREPAIR-B: a prospective cohort study of the impact of germline DNA repair mutations on the outcomes of patients with metastatic castration-resistant prostate cancer. J Clin Oncol.

[4] Lee D.J., Hausler R., Le A.N. (2022). Association of inherited mutations in DNA repair genes with localized prostate cancer. Eur Urol.

[5] Wu Y., Yu H., Li S. (2020). Rare germline pathogenic mutations of DNA repair genes are most strongly associated with grade group 5 prostate cancer. Eur Urol Oncol.

[6] Robinson D., Van Allen E.M., Wu Y.M. (2015). Integrative clinical genomics of advanced prostate cancer. Cell.

[7] Abida W., Cheng M.L., Armenia J. (2019). Analysis of the prevalence of microsatellite instability in prostate cancer and response to immune checkpoint blockade. JAMA Oncol.

[8] Rodrigues D.N., Rescigno P., Liu D. (2018). Immunogenomic analyses associate immunological alterations with mismatch repair defects in prostate cancer. J Clin Invest.

[9] Mottet N, Cornford P, van den Bergh RCN, et al. EAU - EANM - ESTRO - ESUR - ISUP - SIOG guidelines on prostate cancer. https://uroweb.org/guidelines/prostate-cancer/. 10.1016/j.eururo.2024.03.027 38614820

[10] Schaeffer EM, Srinivas S, Antonarakis ES, et al. NCCN clinical practice guidelines in oncology – prostate cancer v3.2022. https://www.nccn.org/guidelines/guidelines-detail?category=1&id=1459.

[11] Vasen HFA, Hes FJ, de Jong MM, et al. Erfelijke tumoren: richtlijnen voor diagnostiek en preventie 2017. 6e druk. Leiden, the Netherlands: Stichting Opsporing Erfelijke Tumoren (STOET)/Vereniging Klinische Genetica Nederland (VKGN); 2017. Update 2022; preliminary version ahead of publication made available by M. Ausems.

[12] Parker C., Castro E., Fizazi K. (2020). Prostate cancer: ESMO clinical practice guidelines for diagnosis, treatment and follow-up. Ann Oncol.

[13] RAND Corporation. Delphi method. 2018. https://www.rand.org/topics/delphi-method.html.

[14] Fitch K., Bernstein S., Aguilar M.D. (2001).

[15] Page E.C., Bancroft E.K., Brook M.N. (2019). Interim results from the IMPACT study: evidence for prostate-specific antigen screening in BRCA2 mutation carriers. Eur Urol.

[16] Bancroft E.K., Page E.C., Brook M.N. (2021). A prospective prostate cancer screening programme for men with pathogenic variants in mismatch repair genes (IMPACT): initial results from an international prospective study. Lancet Oncol.

[17] Segal N., Ber Y., Benjaminov O. (2020). Imaging-based prostate cancer screening among BRCA mutation carriers-results from the first round of screening. Ann Oncol.

[18] Nyberg T., Frost D., Barrowdale D. (2020). Prostate cancer risks for male BRCA1 and BRCA2 mutation carriers: a prospective cohort study. Eur Urol.

[19] Na R., Zheng S.L., Han M. (2017). Germline mutations in ATM and BRCA1/2 distinguish risk for lethal and indolent prostate cancer and are associated with early age at death. Eur Urol.

[20] Carter H.B., Helfand B., Mamawala M. (2019). Germline mutations in ATM and BRCA1/2 are associated with grade reclassification in men on active surveillance for prostate cancer. Eur Urol.

[21] Giri V.N., Hegarty S.E., Hyatt C. (2019). Germline genetic testing for inherited prostate cancer in practice: implications for genetic testing, precision therapy, and cascade testing. Prostate.

[22] Hesterberg A.B., Gordetsky J.B., Hurley P.J. (2021). Cribriform prostate cancer: clinical pathologic and molecular considerations. Urology.

[23] Mandelker D., Donoghue M., Talukdar S. (2019). Germline-focussed analysis of tumour-only sequencing: recommendations from the ESMO Precision Medicine Working Group. Ann Oncol.

[24] Pauley K., Koptiuch C., Greenberg S. (2022). Discrepancies between tumor genomic profiling and germline genetic testing. ESMO Open.

[25] Lincoln S.E., Nussbaum R.L., Kurian A.W. (2020). Yield and utility of germline testing following tumor sequencing in patients with cancer. JAMA Netw Open.

[26] Hussain M., Corcoran C., Sibilla C. (2022). Tumor genomic testing for >4000 men with metastatic castration-resistant prostate cancer in the phase III trial profound (olaparib). Clin Cancer Res.

[27] Mateo J., McKay R., Abida W. (2020). Accelerating precision medicine in metastatic prostate cancer. Nat Cancer.

[28] Cheng H.H., Sokolova A.O., Schaeffer E.M., Small E.J., Higano C.S. (2019). Germline and somatic mutations in prostate cancer for the clinician. J Natl Compr Canc Netw.

[29] Abida W., Armenia J., Gopalan A. (2017). Prospective genomic profiling of prostate cancer across disease states reveals germline and somatic alterations that may affect clinical decision making. JCO Precis Oncol.

[30] Schweizer M.T., Sivakumar S., Tukachinsky H. (2021). Concordance of DNA repair gene mutations in paired primary prostate cancer samples and metastatic tissue or cell-free DNA. JAMA Oncol.

[31] Mateo J., Seed G., Bertan C. (2020). Genomics of lethal prostate cancer at diagnosis and castration resistance. J Clin Invest.

[32] de Bono J., Mateo J., Fizazi K. (2020). Olaparib for metastatic castration-resistant prostate cancer. N Engl J Med.

[33] Slootbeek P.H.J., Kloots I.S.H., Smits M. (2022). Impact of molecular tumour board discussion on targeted therapy allocation in advanced prostate cancer. Br J Cancer.

