# Supplementary material for: Genetic Aspects and Molecular Testing in Prostate Cancer: A Report from a Dutch Multidisciplinary Consensus Meeting
Source: Eur Urol Open Sci. 2023 Jan 25;49:23–31. doi: 10.1016/j.euros.2022.11.011 (PMC9975012; doi:10.1016/j.euros.2022.11.011)

SUPPLEMENTARY MATERIALS

1. (Inter)national recommendations for germline and tumour genetic testing in PCa
2. Scenarios & consensus questions

(Inter)national recommendations for germline and tumour genetic testing in prostate cancer

Guidelines: EAU and ESMO

	Germline testing	Tumour genetic testing
EAU [1]	Consider for:<br>• Men with metastatic Pca<br>• Men with high-risk PCa and family member diagnosed with PCa at age <60 year<br>• Men with multiple family members diagnosed with PCa at age <60 year or a family member who died of Pca<br>• Men with family history of high-risk germline mutations or family history of multiple cancers on same side of family	Should be offered to mCRPC patients for testing of HRR as well as MMR deficiencies or MSI
ESMO [2]	• Recommend for *BRCA2* and other DDR genes associated with cancer predisposition syndromes in patients with a family history of cancer<br>• Consider in all patients with metastatic PCa<br>• Patients with pathogenic mutations in cancer-risk genes identified through tumour testing should be referred for germline testing and genetic counselling	Consider in mCRPC patients for testing of HRR genes and MRR defects or MSI

1. Mottet N, Cornford P, van den Bergh RCN, et al. EAU - EANM - ESTRO - ESUR - ISUP -SIOG guidelines on prostate cancer. Available at: https://uroweb.org/guidelines/prostate-cancer/).
2. Parker C, Castro E, Fizazi K, et al. Prostate cancer: ESMO clinical practice guidelines for diagnosis, treatment and follow-up. Ann Oncol 2020;31:1119-34.

Guidelines: NCCN and Dutch

	Germline testing	Tumour genetic testing
NCCN [1]	Recommend for:<br>• High-risk, very high-risk, regional or metastatic PCa regardless of family history and age of diagnosis<br>• PCa and Ashkenazi Jewish ancestry<br>• PCa and known family history of high-risk germline mutations<br>• PCa and positive family history of cancer<br>• ≥1 first-, second-, or third-degree relative with:<br>• at age ≤50 year: breast, colorectal or endometrial cancer<br>• at any age: male breast cancer, ovarian, exocrine pancreatic, or (very) high-risk, regional or metastatic PCa<br>• ≥1 first-degree relative with PCa at age ≤60 year<br>• ≥2 first-, second-, or third-degree relatives with breast or prostate cancer at any age<br>• ≥3 first- or second-degree relatives with Lynch syndrome-related cancers, especially if diagnosed at age <50 years<br>• PCa and personal history of breast cancer<br>Consider for:<br>• Intermediate-risk PCa and intraductal/cribriform histology<br>• PCa and personal history of some other cancers<br>Genes:<br>• *MLH1, MSH2, MSH6, PMS2, BRCA1, BRCA2, ATM, PALB2, CHEK2* & others depending on clinical context	Recommend:<br>• DDR genes in patients with metastatic PCa (*BRCA1*, *BRCA2*, *ATM*, *PALB2*, *FANCA*, *RAD51D*, *CHEK2*, *CDK12*)<br>• MSI or MMR genes in patients with mCRPC<br><br>Consider:<br>• DDR genes in patients with regional PCa<br>• MSI or MMR genes in patients with regional PCa or mHSPC<br>• TMB testing in patients with mCRPC
Dutch [2]	Consider for patient with PCa (Gleason score ≥7) and:<br>• First- or second-degree relative with breast cancer diagnosed at age ≤50 years, or ovarian cancer, or pancreatic cancer<br>• Ashkenazi Jewish ancestry	None

1. Schaeffer EM, Srinivas S, Antonarakis ES, et al. NCCN Clinical Practice Guidelines in Oncology – Prostate Cancer v3.2022. Available at: https://www.nccn.org/guidelines/guidelines-detail?category=1&id=1459.
2. Vasen HFA, Hes FJ, de Jong MM, et al. Erfelijke tumoren: richtlijnen voor diagnostiek en preventie 2017. 6e druk. Leiden: Stichting Opsporing Erfelijke Tumoren (STOET)/Vereniging Klinische Genetica Nederland (VKGN); 2017. Update 2022; preliminary version ahead of publication made available by M. Ausems.

Scenarios & consensus questions

Scenario 1

45-year-old man, concerned if he has or will get PCa

- No urological signs/symptoms
- PSA <1 ng/ml
- Normal DRE
- Family history:
  - Father died at age 64 of metastatic PCa (diagnosis at age 55, Gleason score 8)
  - Brother diagnosed with high-risk localised PCa (Gleason score 7) at age 50
  - No other cancer in the family

PCa = prostate cancer
PSA = prostate-specific antigen
DRE = digital rectal examination

Question 1

How appropriate do you consider the following options for this patient?

Options	Appropriateness (median score)[1]	Can't judge (%)[2]
No further steps	2.0	5
Referral to clinical geneticist for genetic counselling	4.0*	5
Germline genetic testing for the man	2.0	10
Follow-up by PSA	8.0	8
Follow-up by PSA and mpMRI	2.0	18

[1] Score on a 9-point scale: 1-3 inappropriate (red), 4-6 uncertain or disagreement (yellow), 7-9 appropriate (green)
[2] Total number of respondents=39
*Disagreement: at least one-third of the scores in each of the sections 1-3 and 7-9

Scenario 2 (same case with changes marked in yellow)

45-year-old man, concerned if he has or will get PCa

- No urological signs/symptoms
- PSA <1 ng/ml
- Normal DRE
- Family history:
  - Father died at age 64 of metastatic PCa (diagnosis at age 55, Gleason score 8)
  - Brother diagnosed with high-risk localised PCa (Gleason score 7) at age 50
  - Sister diagnosed with ovarian cancer at the age of 55

PCa = prostate cancer
PSA = prostate-specific antigen
DRE = digital rectal examination

Question 2

How appropriate do you consider the following options for this patient?

Options	Appropriateness (median score)[1]	Can't judge (%)[2]
No further steps	2.0	5
Referral to clinical geneticist for genetic counselling	8.0	5
Germline genetic testing for the man	6.0	10
Follow-up by PSA	8.0	8
Follow-up by PSA and mpMRI	2.0	21

[1] Score on a 9-point scale: 1-3 inappropriate (red), 4-6 uncertain or disagreement (yellow), 7-9 appropriate (green)
[2] Total number of respondents=39

Scenario 3 (same case with changes marked in yellow)

45-year-old man, concerned if he has or will get PCa

- No urological signs/symptoms
- PSA <1 ng/ml
- Normal DRE
- Family history:
  - Father died at age 64 of metastatic PCa (diagnosis at age 55, Gleason score 8)
  - Brother diagnosed with high-risk localised PCa (Gleason score 7) at age 50
  - Sister diagnosed with ovarian cancer at the age of 55 (BRCA2 germline (L)PV)
  - The man is carrier of the same BRCA2 germline (L)PV

PCa = prostate cancer
PSA = prostate-specific antigen
DRE = digital rectal examination
(L)PV = (likely) pathogenic variant

Question 3

How appropriate do you consider the following options for this patient?

Options	Appropriateness (median score)[1]	Can't judge (%)[2]
No further steps	1.0	5
Follow-up by PSA	8.0	8
Follow-up by PSA and mpMRI	4.0*	28

[1] Score on a 9-point scale: 1-3 inappropriate (red), 4-6 uncertain or disagreement (yellow), 7-9 appropriate (green)
[2] Total number of respondents=39
*Disagreement: at least one-third of the scores in each of the sections 1-3 and 7-9

Scenario 4 (same case with changes marked in yellow)

45-year-old man, concerned if he has or will get PCa

- No urological signs/symptoms
- PSA <1 ng/ml
- Normal DRE
- Family history:
  - Father died at age 64 of metastatic PCa (diagnosis at age 55, Gleason score 8)
  - Brother diagnosed with high-risk localised PCa (Gleason score 7) at age 50
  - Sister diagnosed with breast cancer at the age of 45 and an ATM germline (L)PV

PCa = prostate cancer
PSA = prostate-specific antigen
DRE = digital rectal examination
(L)PV = (likely) pathogenic variant

Question 4

How appropriate do you consider the following options for this patient?

Options	Appropriateness (median score)[1]	Can't judge (%)[2]
No further steps	1.0	5
Germline genetic testing for the man[3]	7.0	10
Follow-up by PSA	8.0	8
Follow-up by PSA and mpMRI	3.0	23

[1] Score on a 9-point scale: 1-3 inappropriate (red), 4-6 uncertain or disagreement (yellow), 7-9 appropriate (green)
[2] Total number of respondents=39
[3] Especially important for the female relatives of the man

Scenario 5

66-year-old man

- Diagnosed with low-risk PCa (PSA 5.4 ng/ml, Gleason score 6 on targeted biopsy, cT2a)
- ECOG PS: 0
- Family history:
  - Brother 1: diagnosis high-risk localised PCa at age 58 (PSA 18 ng/ml, Gleason score 8, cT2c)
  - Brother 2: diagnosis de novo mHSPC at age 67 (PSA 182 ng/ml, Gleason score 8, and 5 bone metastases), carrier of a BRCA2 tumour (L)PV

ECOG PS: Eastern Cooperative Oncology Group performance status
(L)PV = (likely) pathogenic variant
mHSPC = metastatic hormone-sensitive prostate cancer

Question 5

How appropriate do you consider the following options for this patient?

Options	Appropriateness (median score)[1]	Can't judge (%)[2]
Referral to clinical geneticist & germline genetic testing	3.0	5
Tumour genetic testing	2.0	8
Active surveillance	8.0	8
Active treatment (surgery/radiotherapy)	5.0	21

[1] Score on a 9-point scale: 1-3 inappropriate (red), 4-6 uncertain or disagreement (yellow), 7-9 appropriate (green)
[2] Total number of respondents=39

Scenario 6 (same case with changes marked in yellow)

66-year-old man

- Diagnosed with low-risk PCa (PSA 5.4 ng/ml, Gleason score 6 on targeted biopsy, cT2a)
- ECOG PS: 0
- Family history:
  - Brother 1: diagnosis high-risk localised PCa at age 58 (PSA 18 ng/ml, Gleason score 8, cT2c)
  - Brother 2: diagnosis de novo mHSPC at age 67 (PSA 182 ng/ml, Gleason score 8, and 5 bone metastases), carrier of a BRCA2 germline (L)PV

ECOG PS: Eastern Cooperative Oncology Group performance status
(L)PV = (likely) pathogenic variant
mHSPC = metastatic hormone-sensitive prostate cancer

Question 6

How appropriate do you consider the following options for this patient?

Options	Appropriateness (median score)[1]	Can't judge (%)[2]
Referral to clinical geneticist & germline genetic testing	8.0	8
Tumour genetic testing	2.0	15
Active surveillance	7.0	13
Active treatment (surgery/radiotherapy)	5.5	28

[1] Score on a 9-point scale: 1-3 inappropriate (red), 4-6 uncertain or disagreement (yellow), 7-9 appropriate (green)
[2] Total number of respondents=39

Scenario 7 (same case with changes marked in yellow)

66-year-old man

- Diagnosed with low-risk PCa (PSA 5.4 ng/ml, Gleason score 6 on targeted biopsy, cT2a)
- ECOG PS: 0
- Family history:
  - Brother 1: diagnosis high-risk localised PCa at age 58 (PSA 18 ng/ml, Gleason score 8, cT2c)
  - Brother 2: diagnosis de novo mHSPC at age 67 (PSA 182 ng/ml, Gleason score 8, and 5 bone metastases), carrier of a BRCA2 germline (L)PV
- Patient is also carrier of the same BRCA2 germline (L)PV

ECOG PS: Eastern Cooperative Oncology Group performance status
(L)PV = (likely) pathogenic variant
mHSPC = metastatic hormone-sensitive prostate cancer

Question 7

How appropriate do you consider the following options for this patient?

Options	Appropriateness (median score)[1]	Can't judge (%)[2]
Active surveillance	6.0	15
Active treatment (surgery/radiotherapy)	7.0	23

[1] Score on a 9-point scale: 1-3 inappropriate (red), 4-6 uncertain or disagreement (yellow), 7-9 appropriate (green)
[2] Total number of respondents=39

Scenario 8 (same case with changes marked in yellow)

66-year-old man

- Diagnosed with low-risk PCa (PSA 5.4 ng/ml, Gleason score 6 on targeted biopsy, cT2a)
- ECOG PS: 0
- Family history:
  - Brother 1: diagnosis high-risk localised PCa at age 58 (PSA 18 ng/ml, Gleason score 8, cT2c)
  - Brother 2: diagnosis de novo mHSPC at age 67 (PSA 182 ng/ml, Gleason score 8, and 5 bone metastases), carrier of a BRCA2 germline (L)PV
- Patient is not a carrier of the same BRCA2 germline (L)PV

ECOG PS: Eastern Cooperative Oncology Group performance status
(L)PV = (likely) pathogenic variant
mHSPC = metastatic hormone-sensitive prostate cancer

Question 8

How appropriate do you consider the following options for this patient?

Options	Appropriateness (median score)[1]	Can't judge (%)[2]
Active surveillance	8.0	13
Active treatment (surgery/radiotherapy)	3.0	26

[1] Score on a 9-point scale: 1-3 inappropriate (red), 4-6 uncertain or disagreement (yellow), 7-9 appropriate (green)
[2] Total number of respondents=39

Scenario 9

64-year-old man, diagnosed with M0 PCa

- ECOG Performance Status: 0
- No relevant comorbidities
- No relevant family history of cancer

ECOG: Eastern Cooperative Oncology Group

Question 9

When is germline genetic testing in this patient recommended? [multiple answers allowed]

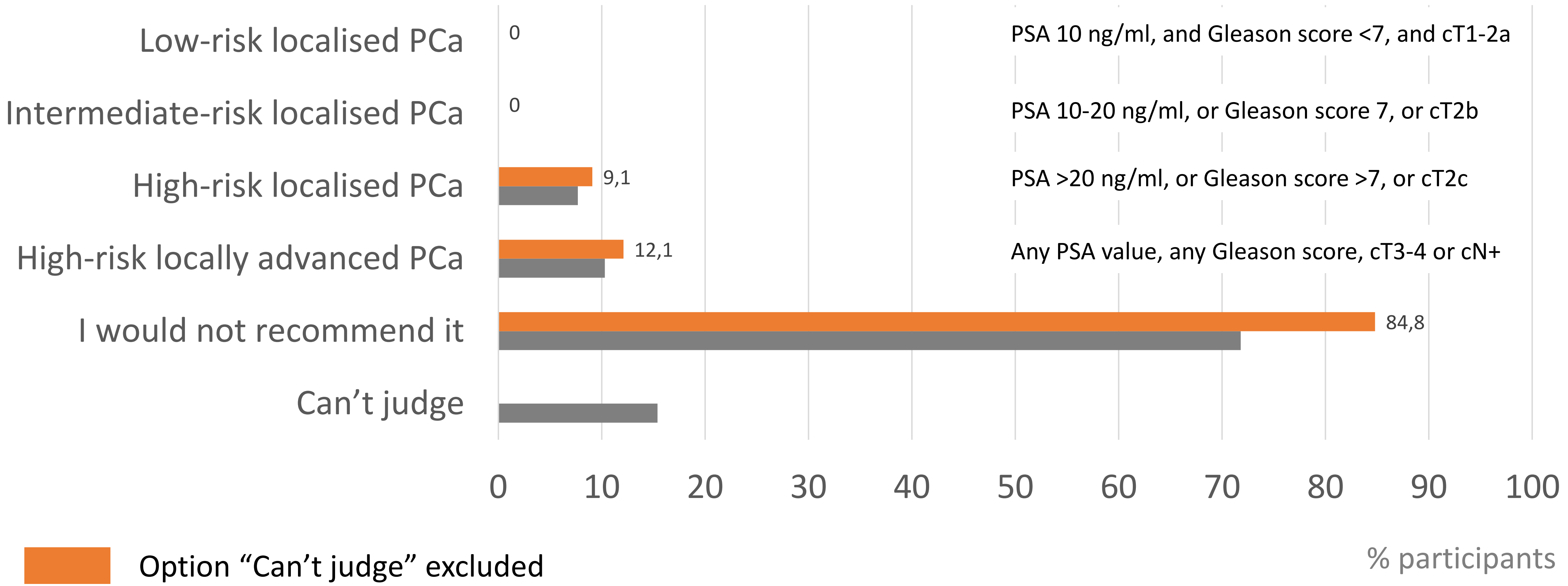

Question 10

When is tumour genetic testing in this patient recommended? [multiple answers allowed]

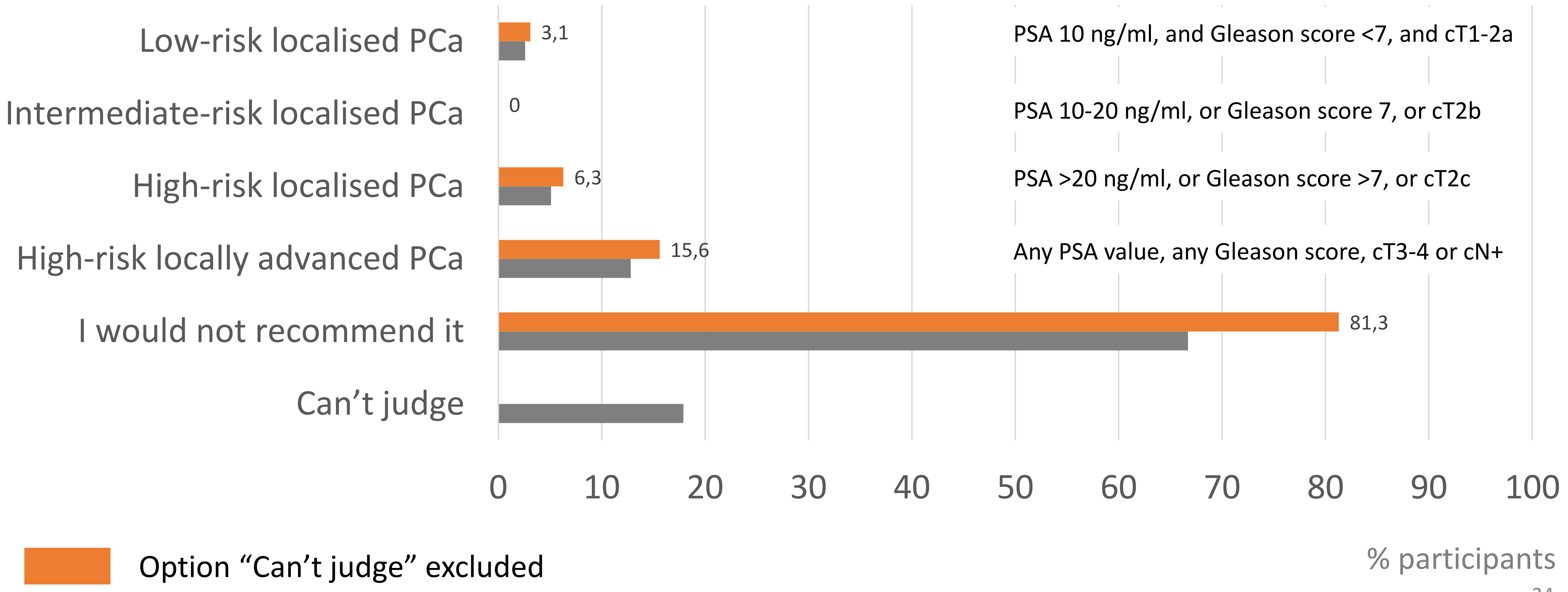

Question 11

How appropriate do you consider germline genetic testing in patients with M0 PCa in the following situations?

Situations	Appropriateness (median score)	Can't judge (%)
≥cT3 stage	2.0	15
cN+	2.0	15
Gleason score 7	2.0	15
Gleason score 8-10	3.0	15
Intraductal/cribriform pathology	2.0	21
PSA >20 ng/ml	2.0	21
PCa family history	5.5	18
Other tumours in the family (pancreas, ovarium, breast at age <50 year)	7.5	13

Question 12

How appropriate do you consider tumour genetic testing in patients with M0 PCa in the following situations?

Situations	Appropriateness (median score)	Can't judge (%)
≥cT3 stage	2.0	23
cN+	2.0	26
Gleason score 7	1.0	26
Gleason score 8-10	3.0	26
Intraductal/cribriform pathology	2.0	31
PSA >20 ng/ml	2.0	31
PCa family history	2.0	23
Other tumours in the family (pancreas, ovarium, breast at age <50 year)	3.0*	23

*Disagreement: at least one-third of the scores in each of the sections 1-3 and 7-9

Scenario 10

70-year old man with metastatic hormone-sensitive PCa (mHSPC)

Question 13

How appropriate do you consider germline testing in this patient with mHSPC in the following situations?

Situations	Appropriateness (median score)[1]	Can't judge (%)[2]
De novo, M1a	2.0	26
De novo, visceral metastases	3.0	26
De novo, high-volume (bone)	3.0	26
De novo, low-volume	2.0	33
Recurrent, M1a	3.0	26
Recurrent, visceral metastases	3.0	26
Recurrent, high-volume	3.0	26
Recurrent, low-volume	3.0	26

[1] Score on a 9-point scale: 1-3 inappropriate (red), 4-6 uncertain or disagreement (yellow), 7-9 appropriate (green)
[2] Total number of respondents=39

Question 14

How appropriate do you consider tumour genetic testing in this patient with mHSPC in the following situations?

Situations	Appropriateness (median score)[1]	Can't judge (%)[2]
De novo, M1a	3.0	28
De novo, visceral metastases	5.0	31
De novo, high-volume (bone)	4.0*	31
De novo, low-volume	4.0	33
Recurrent, M1a	3.5	33
Recurrent, visceral metastases	6.0	31
Recurrent, high-volume	5.0	31
Recurrent, low-volume	4.0	33

[1] Score on a 9-point scale: 1-3 inappropriate (red), 4-6 uncertain or disagreement (yellow), 7-9 appropriate (green)

[2] Total number of respondents=39

*Disagreement: at least one-third of the scores in each of the sections 1-3 and 7-9

Question 15

How important is tumour genetic testing in patients with high-volume mHSPC (CHAARTED criteria) for:

Aims	Importance (median score)[1]	Can't judge (%)[2]
Detecting increased risk of other types of cancer in the patient	3.0	26
Therapeutic consequences for the patient	6.5	23
Detecting increased risk of other types of cancer in the patient's family	3.5*	23

[1] Score on a 9-point scale: 1-3 (very) unimportant (red), 4-6 uncertain or disagreement (yellow), 7-9 (very) important (green)
[2] Total number of respondents=39
*Disagreement: at least one-third of the scores in each of the sections 1-3 and 7-9

Question 16

How appropriate is further germline genetic testing in patients with high-volume mHSPC (CHAARTED criteria) and:

	Appropriateness (median score)[1]	Can't judge (%)[2]
BRCA1/2 tumour (L)PV	8.0	18
Non-BRCA1/2 tumour (L)PV (e.g. ATM, CHEK2)	7.0	21

[1] Score on a 9-point scale: 1-3 inappropriate (red), 4-6 uncertain or disagreement (yellow), 7-9 appropriate (green)
[2] Total number of respondents=39

Question 17

Has the presence of a BRCA2 tumour (L)PV in a patient with de novo, low-volume mHSPC impact on the choice of your upfront treatment?

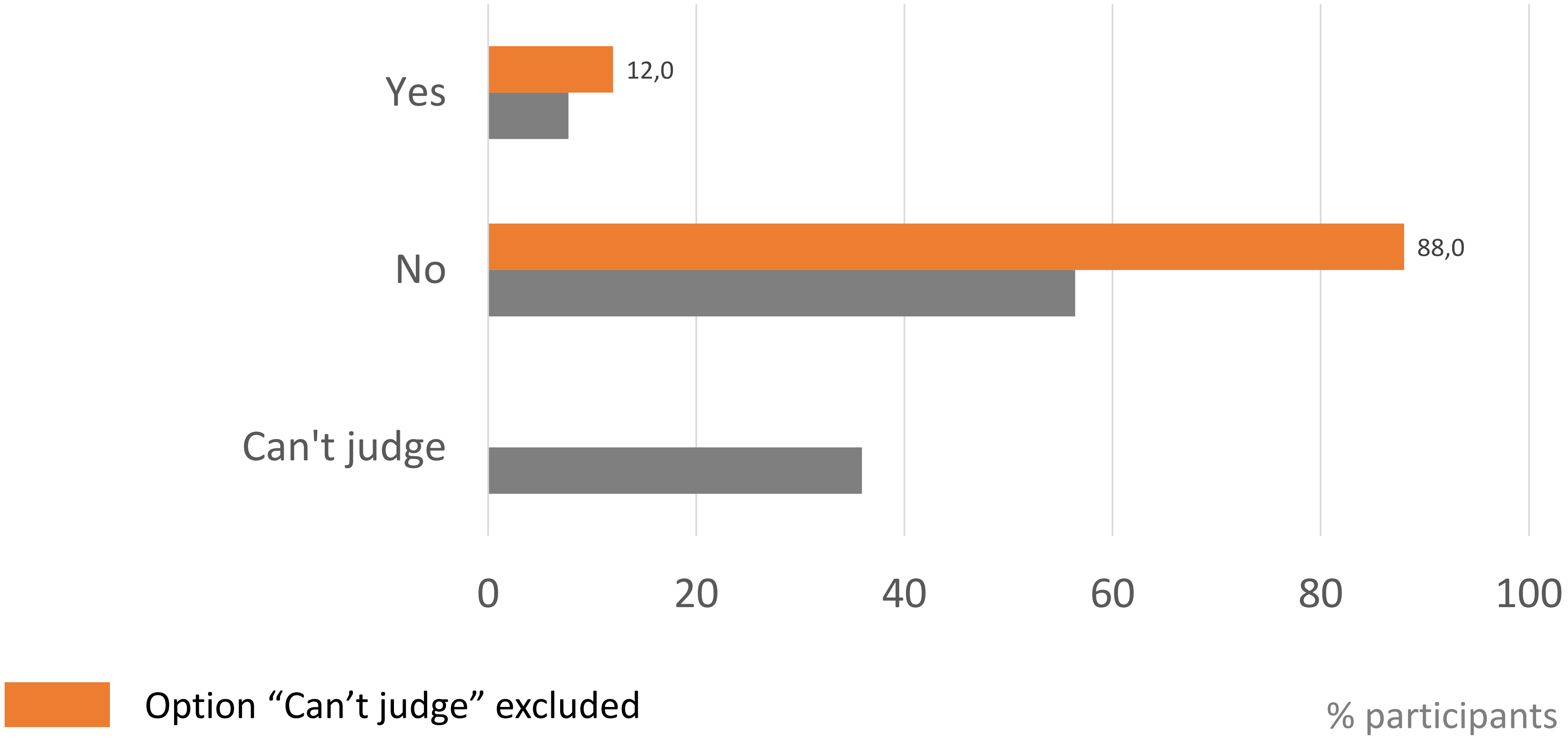

Question 18

Has the presence of a tumour (L)PV in a non-BRCA gene (e.g. CHEK2) in a patient with de novo, low-volume mHSPC impact on the choice of your upfront treatment?

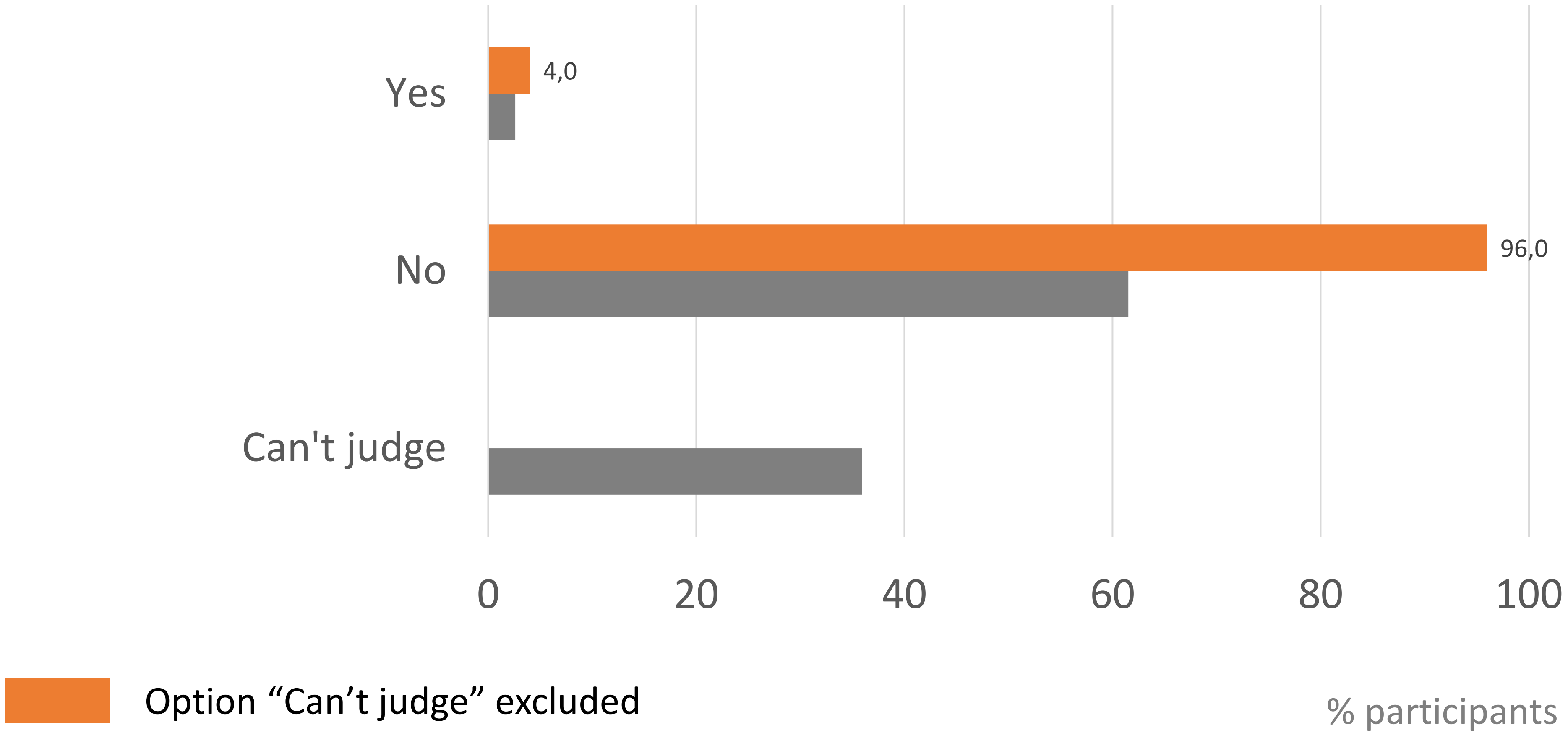

Question 19

Has the presence of a BRCA2 tumour (L)PV in a patient with de novo, high-volume mHSPC impact on the choice of your upfront treatment?

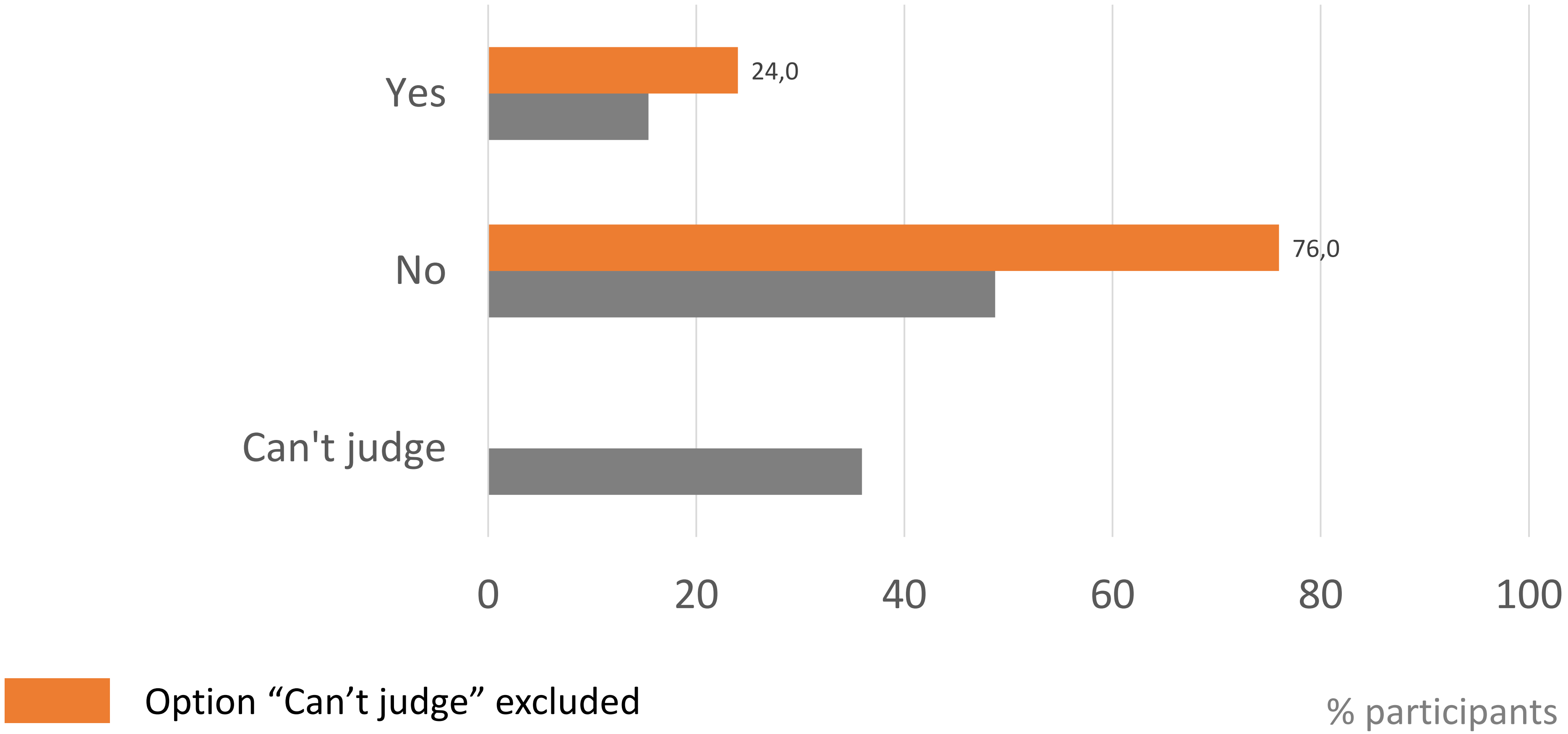

Question 20

In case a tumour genetic test is recommended for a patient with a primary diagnosis of mHSPC (de novo), then the following source of tissue is preferred:

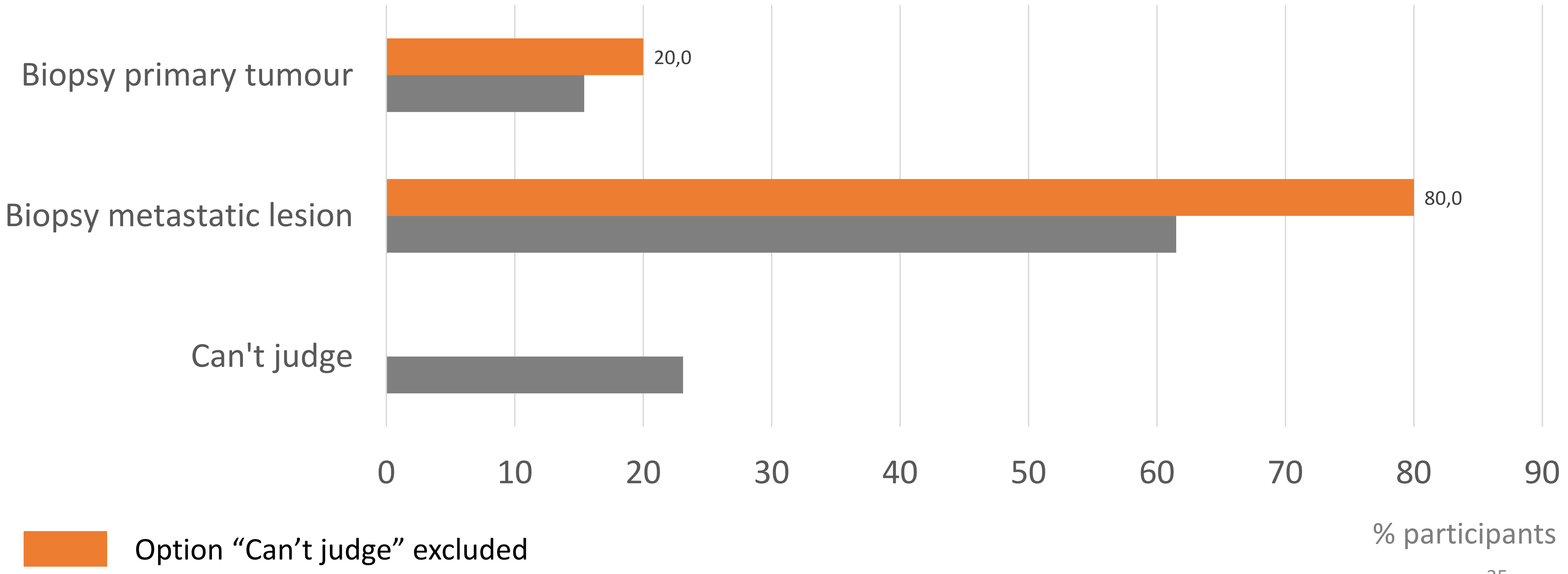

Question 21

In case a tumour genetic test is recommended for a patient with progressive disease following curative treatment, then the following source of tissue is preferred:

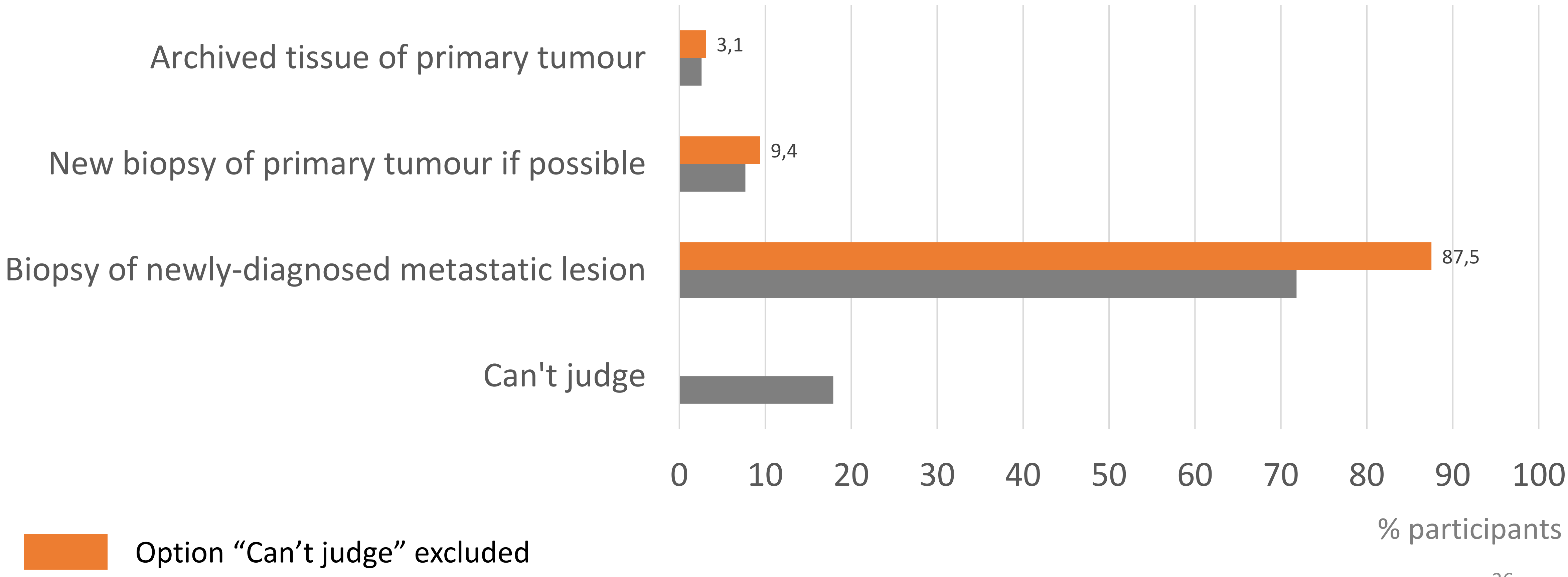

Scenario 11

Progression to mCRPC

Question 22

How appropriate is genetic testing to identify actionable variants in a mCRPC patient?

Options	Appropriateness (median score)[1]	Can't judge (%)[2]
Germline genetic testing	4.0	21
Tumour genetic testing	8.5	18

[1] Score on a 9-point scale: 1-3 (very) unimportant (red), 4-6 uncertain or disagreement (yellow), 7-9 (very) important (green)
[2] Total number of respondents=39

Question 23

From a clinical perspective, how appropriate do you consider tumour genetic testing to identify actionable variants in patients with mCRPC if they have the following characteristics:

Characteristic	Appropriateness (median score)[1]	Can't judge (%)[2]
Young age of patient	8.0	28
Stage at initial diagnosis: mHSPC	7.0	36
Stage at initial diagnosis: localised Pca	6.0	36
Presence of visceral or liver mestastases	8.0	33
Only bone metastases (high-volume)	7.0	36
Duration response to ADT <1 jaar	7.0	39
Duration response to ADT >1 jaar	6.0	39
Gleason score 8-10	8.0	36
Primary resistance to therapy	7.0	44
Presence of PCa in family	7.0	31
Presence of familial PCa	8.0	28

[1] Score on a 9-point scale: 1-3 inappropriate (red), 4-6 uncertain or disagreement (yellow), 7-9 appropriate (green)

[2] Total number of respondents=39

Question 24

How important is further germline genetic testing in a mCRPC patient having a BRCA1/2 tumour (L)PV?

	Importance (median score)[1]	Can't judge (%)[2]
Germline genetic testing	8.0	15

[1] Score on a 9-point scale: 1-3 (very) unimportant (red), 4-6 uncertain or disagreement (yellow), 7-9 (very) important (green)
[2] Total number of respondents=39

Question 25

In a patient with mCRPC, fit enough to receive several therapy lines and not yet treated with novel hormonal agents (NHA) or chemotherapy in the mHSPC, I would recommend tumour genetic testing in daily clinical practice:

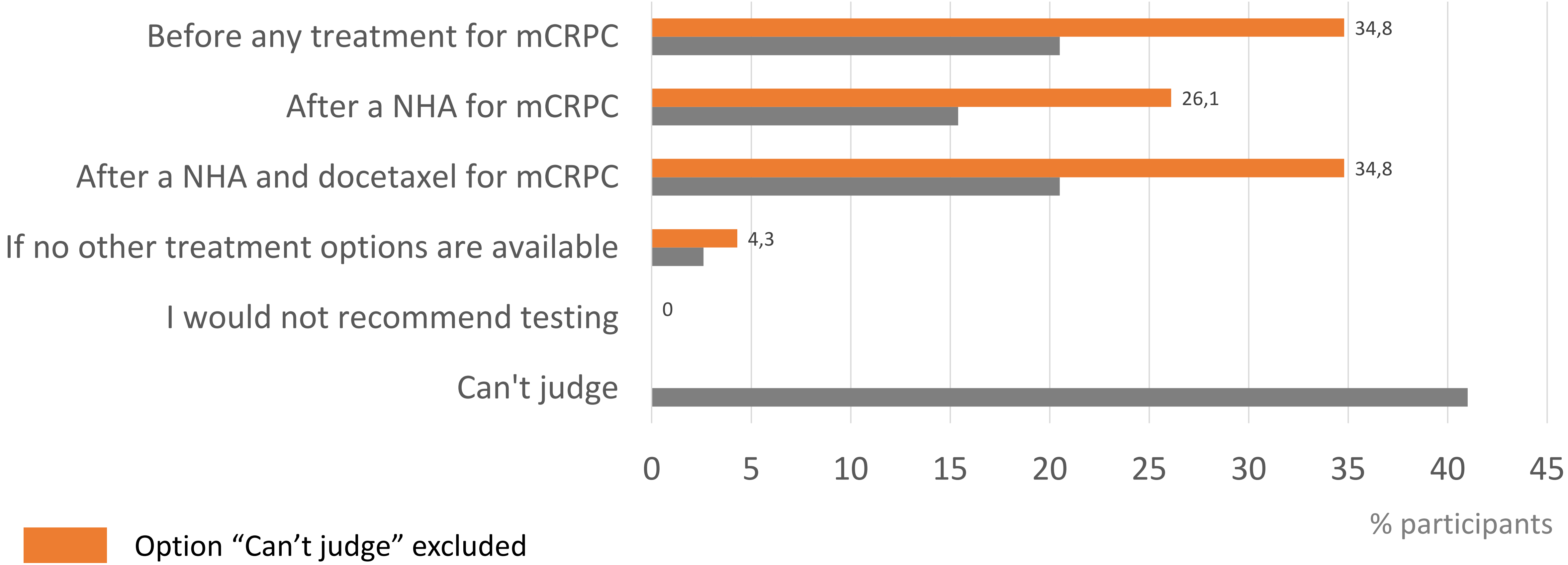

Question 26

In a patient with mCRPC, fit enough to receive several therapy lines and not yet treated with novel hormonal agents (NHA) or chemotherapy in the mHSPC, I would recommend tumour genetic testing <u>in an experimental setting:</u>

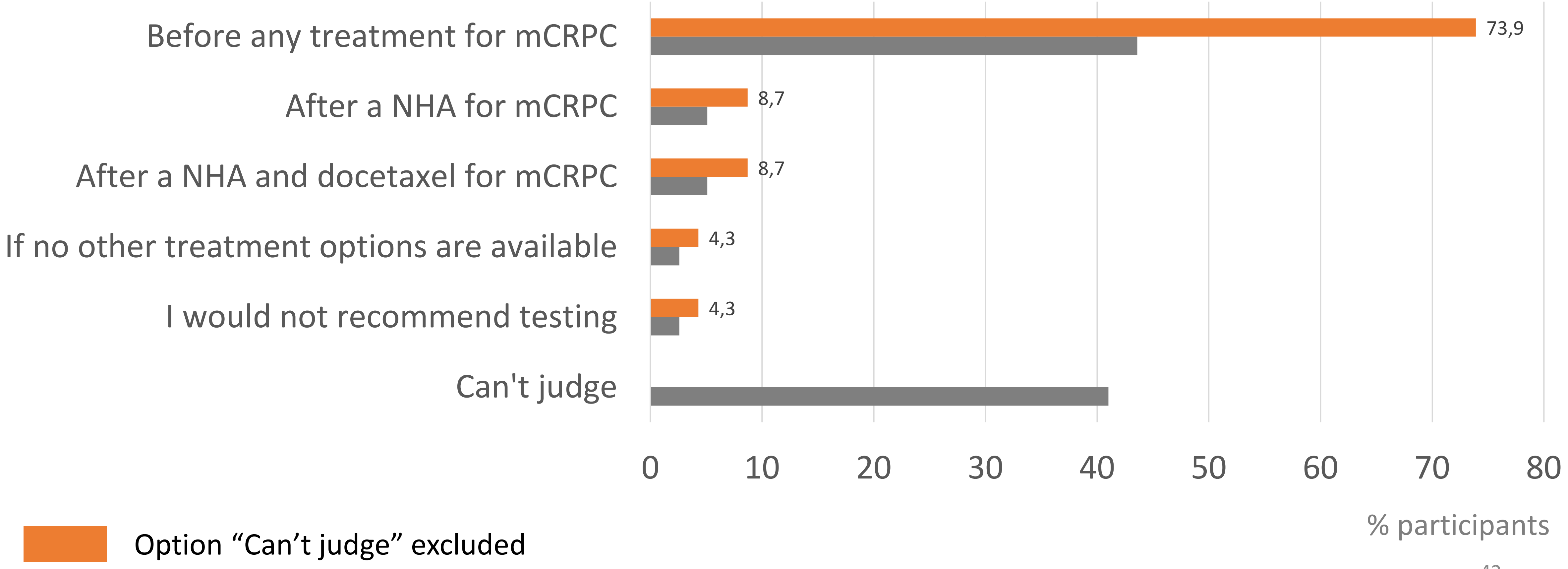

Question 27

How appropriate do you consider the following tissue sources if tumour genetic testing is recommended for an mCRPC patient?

Source	Importance (median score)[1]	Can't judge (%)[2]
Archived diagnostic tissue of the primary tumour	6.0	18
Most recent archived tumour tissue	7.0	18
New biopsy of prostate if feasible	7.0	23
New biopsy bone metastasis	8.0	21
New biopsy lymph node metastasis	8.0	21
New biopsy visceral metastasis	8.0	23

[1] Score on a 9-point scale: 1-3 (very) unimportant (red), 4-6 uncertain or disagreement (yellow), 7-9 (very) important (green)
[2] Total number of respondents=39

Question 28

What are the minimum requirements for tumour percentage of an archived or newly-obtained biopsy for performing somatic tumour testing?

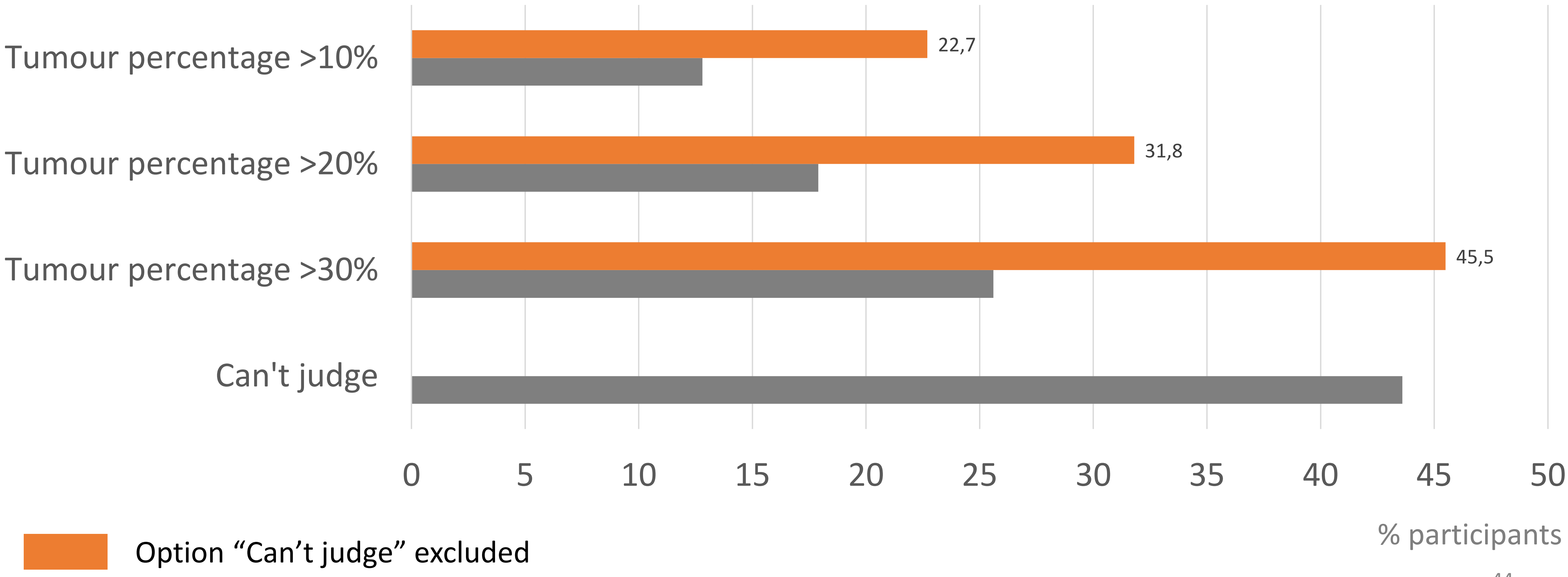

Question 29

What are the minimum requirements for biopsy age if archived tissue is used to perform somatic tumour testing?

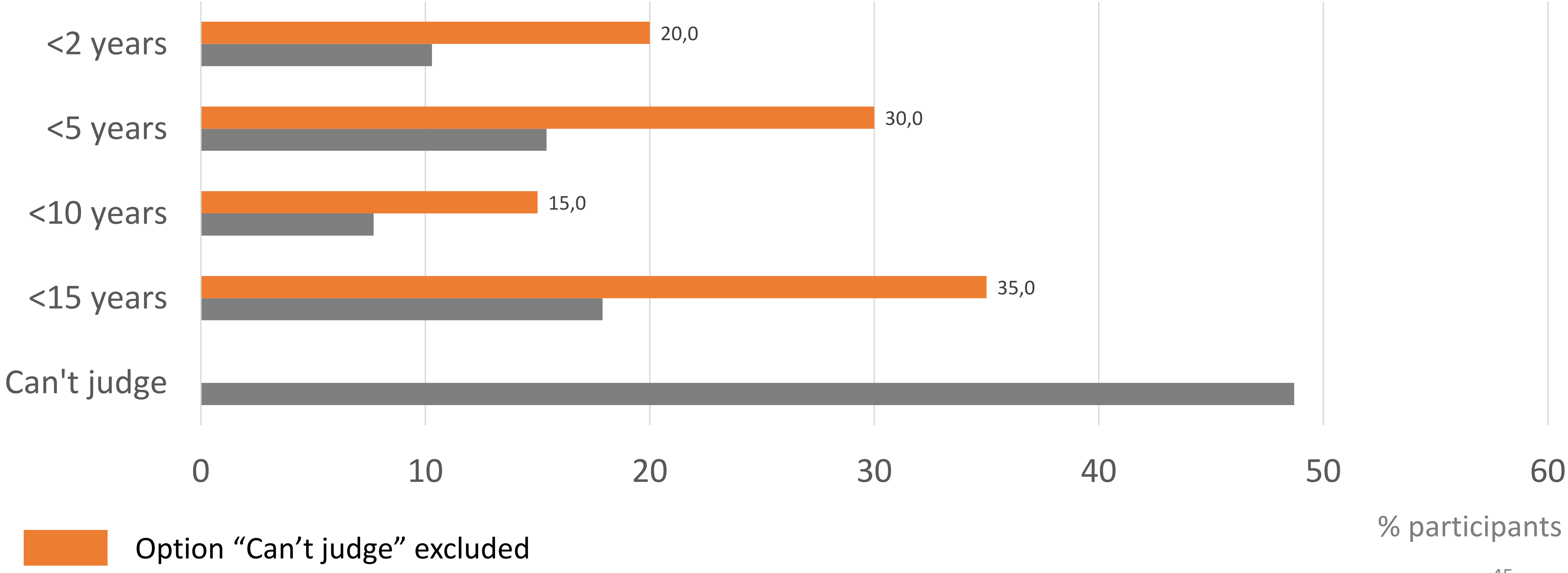

Question 30

Which are the most relevant gene panels to use for somatic testing in a patient with mCRPC for clinical decision making in daily practice? [multiple answers allowed]

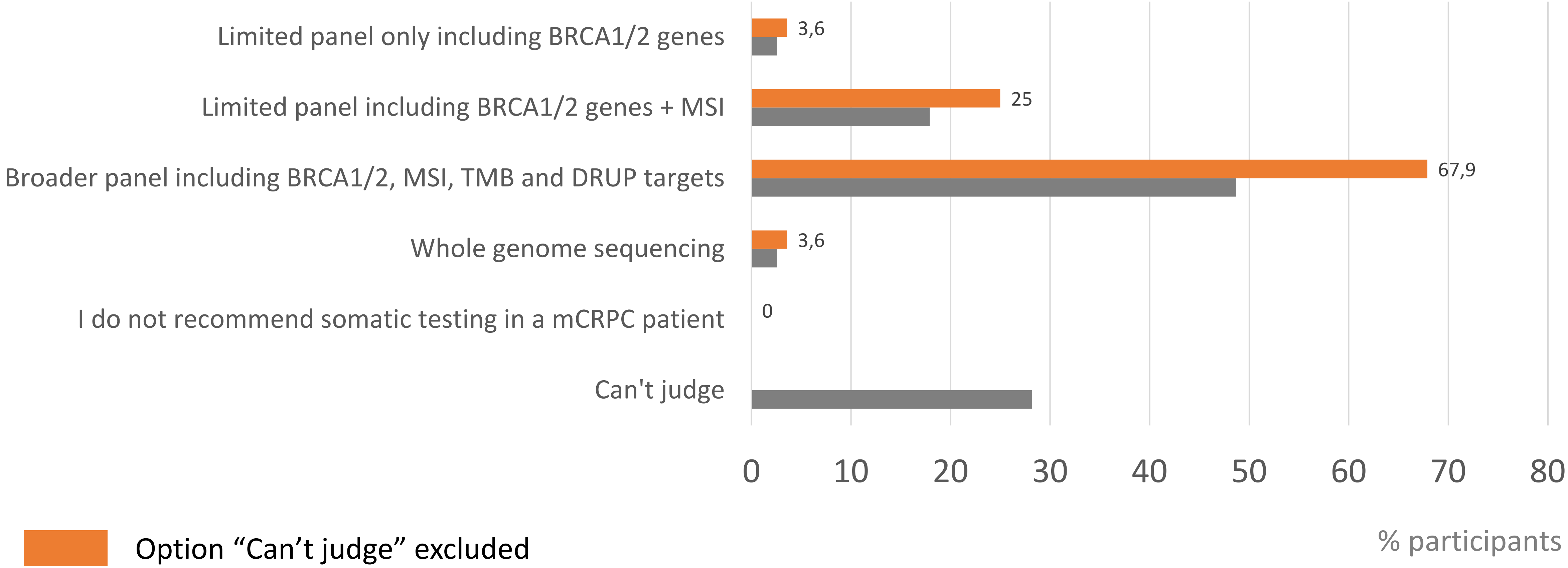

Question 31

Which are the most relevant gene panels to use for somatic testing in a patient with mCRPC for research purposes? [multiple answers allowed]

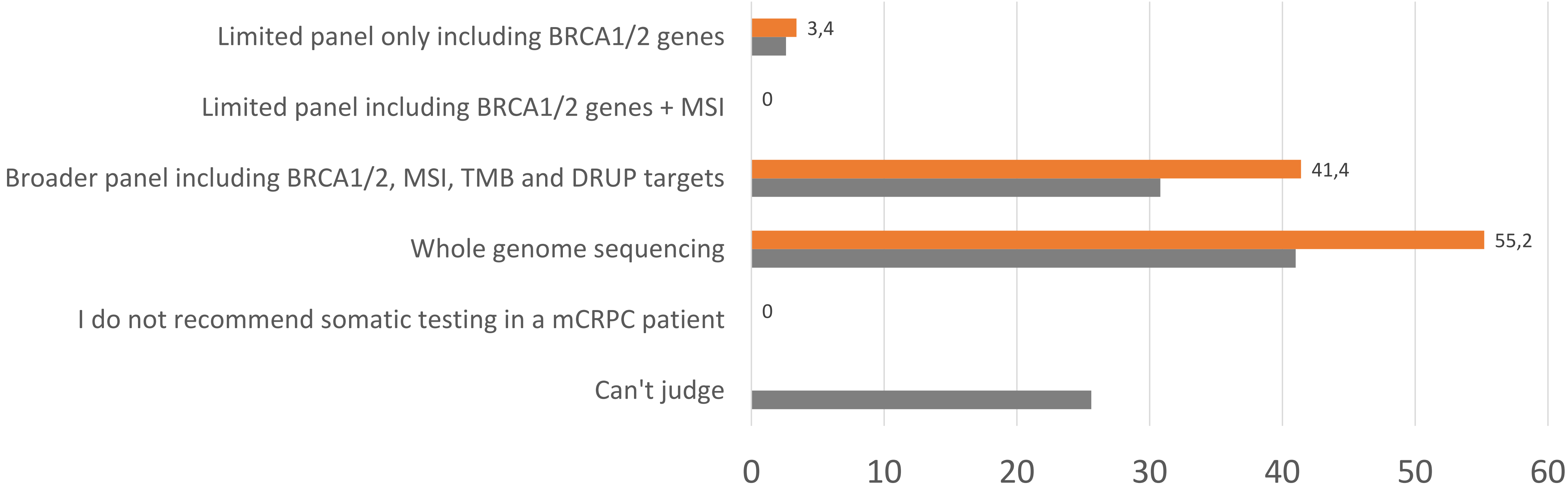

Option "Can't judge" excluded

% participants

Question 32

Where should tumour genetic testing be carried out?

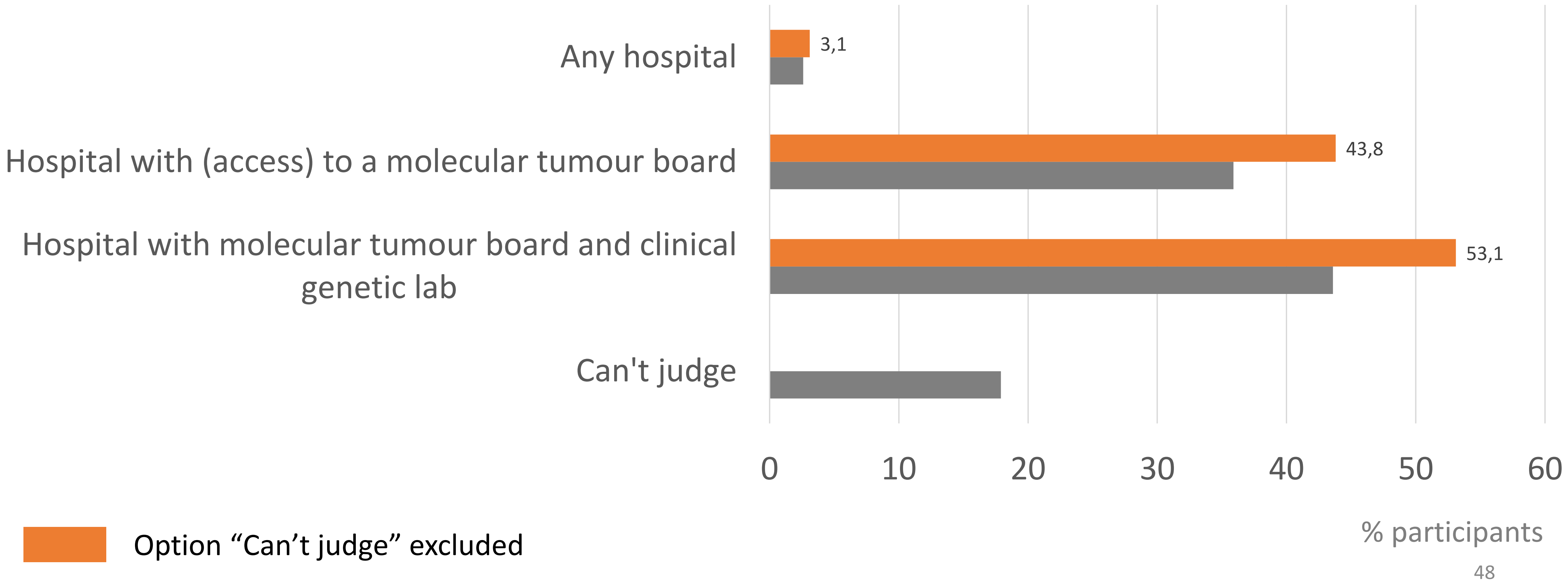

Question 33

When would you use a PARPi in a patient with mCRPC and a BRCA1/2 germline and/or tumour (L)PV?

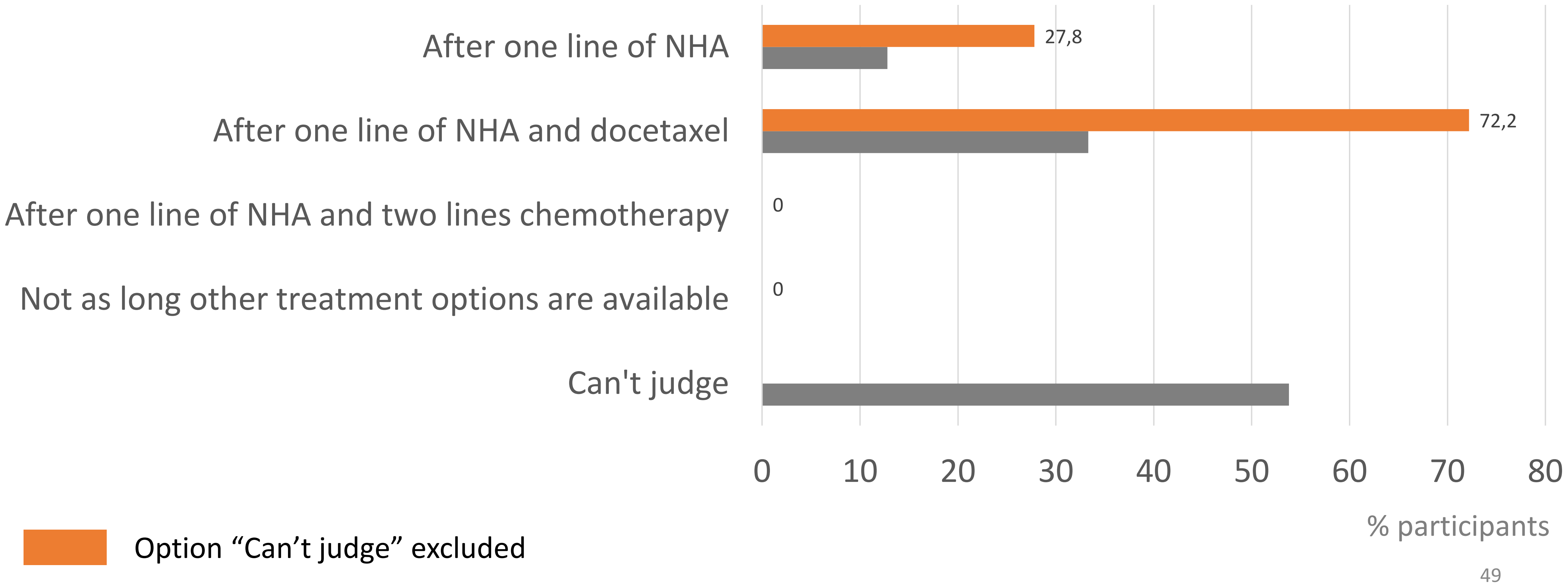

Question 34

When would you use an immune checkpoint inhibitor in a patient with mCRPC and a MMR (L)PV or MSI?

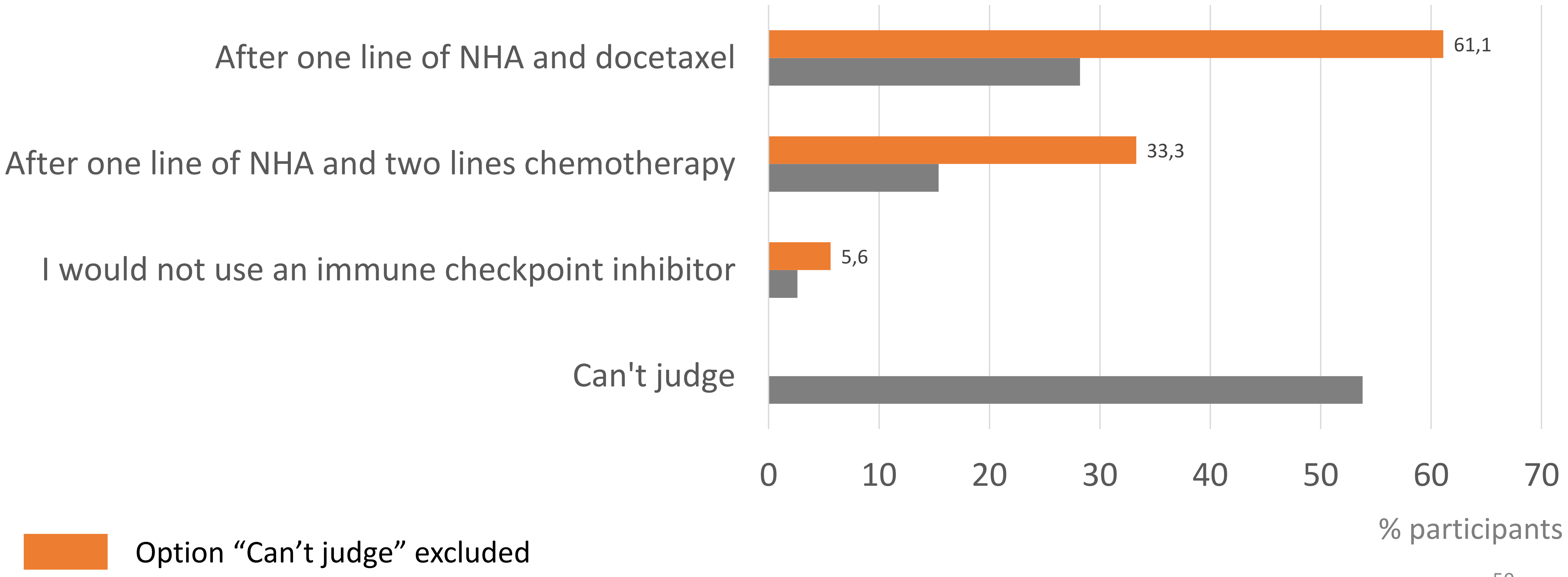

Question 35

Do you recommend platinum-based chemotherapy to patients with mCRPC and a BRCA1/2 tumour and/or germline (L)PV before a possible treatment with a PARPi?

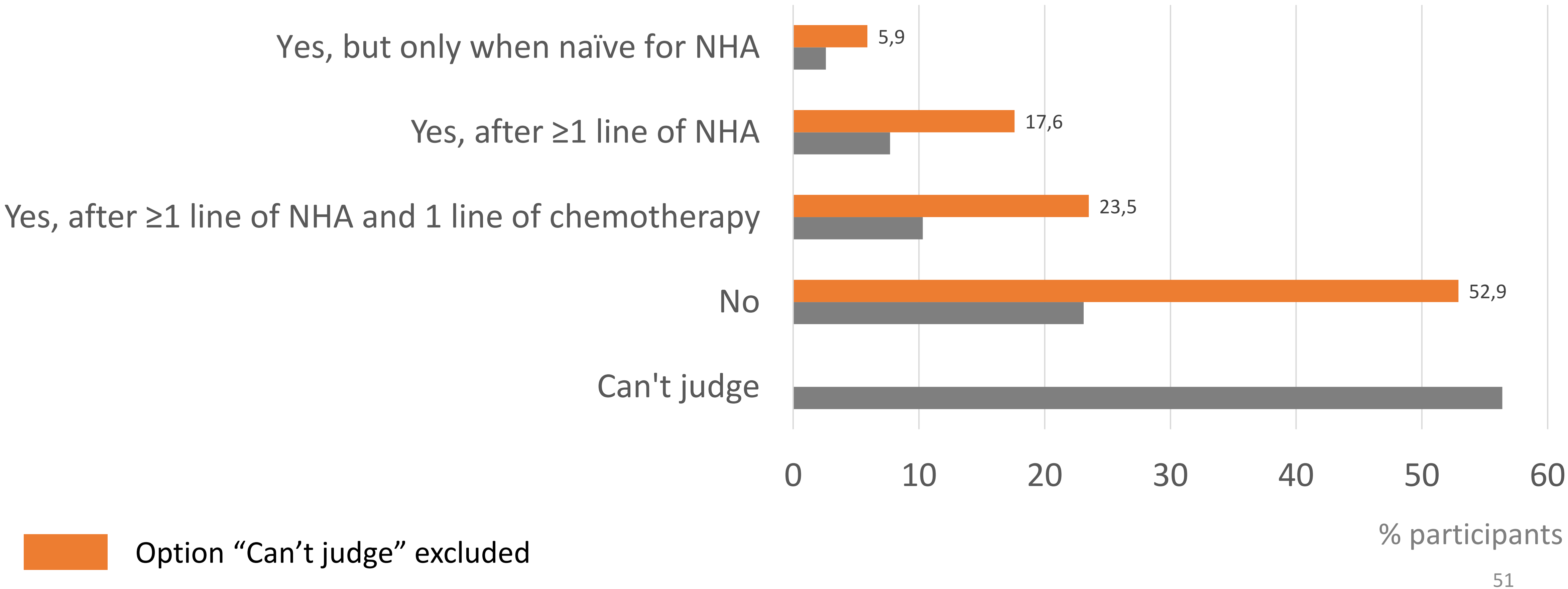

Question 36

Do you recommend platinum-based chemotherapy to patients with mCRPC and a BRCA1/2 tumour and/or germline (L)PV after progression with a PARPi?

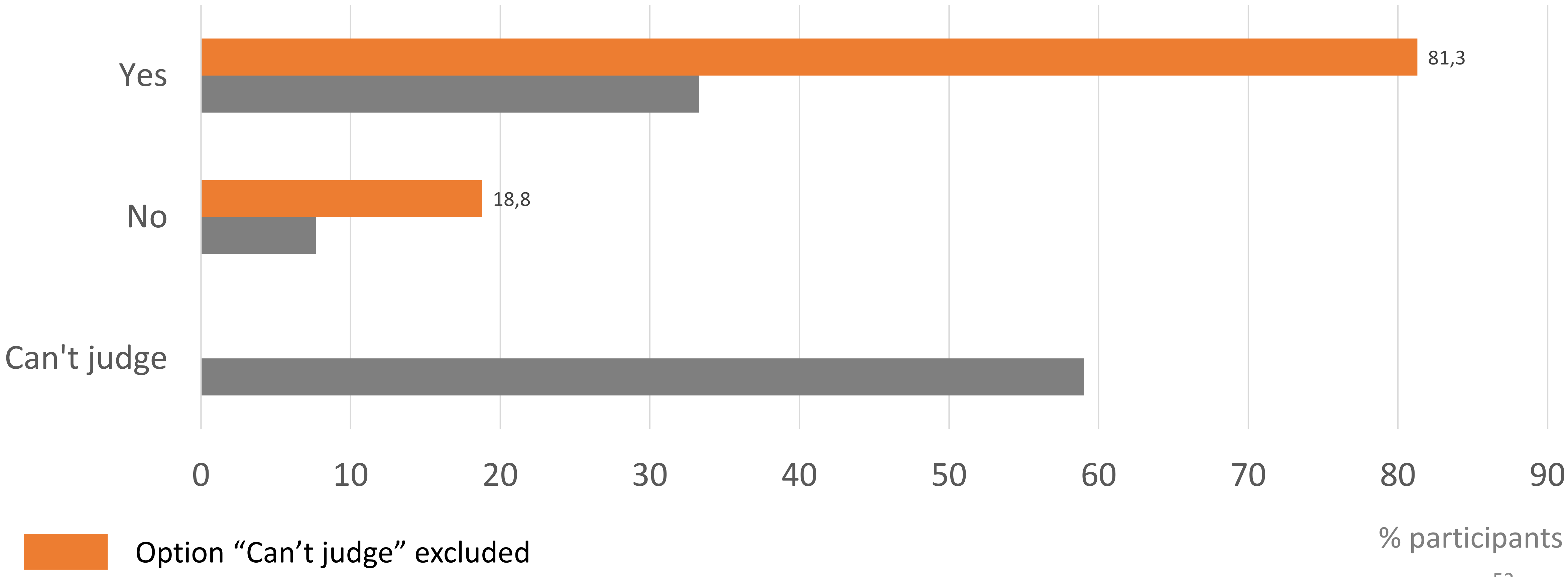

Supplement: Supplementary Data 1 [file mmc1.pdf]
